# Fusidic Acid Reverses Chemoresistance in Breast Cancer via Targeting DDX6 to Downregulate GSK‐3β/β‐Catenin Signaling

**DOI:** 10.1002/advs.202504680

**Published:** 2025-08-04

**Authors:** Xiaxia Fan, Dan Guo, Songtao Li, Jinmiao Tian, Chaotong Zhang, Zhuoyu Li

**Affiliations:** ^1^ Key Laboratory of Chemical Biology and Molecular Engineering of the National Ministry of Education Institute of Biotechnology Shanxi University Taiyuan 030031 China

**Keywords:** fusidic acid, chemoresistance, DDX6, GSK‐3β/β‐catenin signaling, HSC70

## Abstract

ABC transporter protein‐mediated drug efflux is a significant contributor to induced resistance in breast cancer (BC). Novel therapies are therefore urgently needed to thwart chemoresistance. Herein, it is demonstrated that fusidic acid (FA) reduces the expression of ABC transporter proteins MRP1, P‐gp, and BCRP, promotes the in vivo accumulation of agents, and exertes chemosensitizing effects. Further, DDX6 is identified as a direct target of FA by DARTS and biotin pull‐down. Mechanistically, the results indicates that FA directly binds to H378 of DDX6, promotes its degradation, and downregulates its downstream p‐GSK‐3β, which in turn enhances the phosphorylation and ubiquitin‐dependent hydrolysis of β‐catenin S33/37/45/T41, blocks pro‐resistance β‐catenin signaling, and further prevents chemoresistance. The inhibitory effect of FA on chemoresistance is blocked following the knockout of DDX6 using CRISPR/Cas9. More importantly, FA also enhances the interaction between DDX6 and HSC70, while it facilitates DDX6 degradation via chaperone‐mediated autophagy (CMA), thereby impairing the GSK‐3β/β‐catenin axis to enhance the efficacy of FA. Consistently, the effect of FA in combating chemoresistance by targeting DDX6 is evident in a xenograft mouse model. These findings reveal a direct protein target and molecular mechanism for FA to reverse chemoresistance, thus supporting the further development of FA as a chemosensitizing agent.

## Graphical abstract

FA directly binds to residue H378 of DDX6 and promotes its interaction with HSC70, leading to CMA degradation of DDX6, thereby impairing GSK‐3β/β‐catenin signaling, inhibiting ABC transporter proteins, and enhancing chemosensitivity.

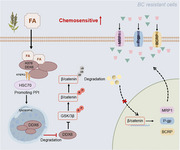

## 1 Introduction

Breast cancer (BC) is the leading cause of cancer‐related deaths in the women population.^[^ ^1^ ^]^ Currently, the main treatment for BC is surgery combined with radiotherapy. Adriamycin (ADR) and taxol (TAX) are the first‐line chemotherapeutic agents for clinical treatment, but the emergence of chemoresistance frequently results in treatment failure or tumor recurrence. Although various attempts have been made to restore the sensitivity of the available chemotherapeutic drugs and to overcome the resistance, the results are still unsatisfactory. Resistance can be endogenous (de novo) or acquired (treatment‐induced), and drivers may be increased drug efflux, decreased intracellular drug uptake, altered drug targeting and dysregulation of apoptosis, epithelial‐mesenchymal transition (EMT), and the presence of stem cells.^[^ ^2^, ^3^ ^]^ The most common cause of triggering resistance is the overexpression and translocation of the ATP‐binding cassette (ABC) transporter proteins, thereby mediating the efflux of drugs and harmful substances in cancer cells and healthy tissues.^[^ ^4^, ^5^ ^]^ Widely studied ABC transporter proteins include multidrug resistance protein 1 (MDR1, also known as P‐gp and ABCB1), MDR‐associated protein 1 (MRP1, also known as ABCC1), and breast cancer resistance protein (BCRP, also known as ABCG2).^[^ ^6^, ^7^ ^]^ These transporters not only mediate unidirectional movement across cellular membranes for a diverse array of substances but also play crucial roles in regulating ion homeostasis along with exogenous compounds and other small molecules within biological systems.^[^ ^8^, ^9^, ^10^, ^11^ ^]^ Hence, MRP1, P‐gp, and BCRP are key factors in the development of chemoresistance. Inhibition of these transport proteins is a possible strategy to reverse chemoresistance.

Fusidic acid (FA) is a triterpenoid that has been shown to possess a wide range of pharmacological activities in vivo and in vitro, including antibacterial, antitumor, anti‐inflammatory, and antiviral activities.^[^ ^12^ ^]^ Specifically, several derivatives of fusidic acid exhibit cytotoxicity in vitro against a variety of tumor cell lines, such as cervical cancer HeLa, multidrug‐resistant oral epidermoid carcinoma KBV, glioma U87, gastric carcinoma MKN45, and colon cancer HT‐29. The mechanism of action includes the reduction of newly synthesized proteins, resulting in the blockage of cells at the G0/G1 phases of the cell cycle, which leads to apoptosis induction.^[^ ^13^, ^14^ ^]^ Furthermore, FA acts as an inhibitor of p53‐related protein kinase (PRPK), and when combined with 5‐fluorouracil (5‐FU), it prevents mice's colon cancer from metastasizing to their lungs.^[^ ^15^ ^]^ These findings support the potential application value of FA in the antitumor field, especially in the development of novel antitumor drugs. We pre‐extracted terpenoids from quinoa, and mass spectrometry identification revealed that the main active ingredient was FA.^[^ ^16^ ^]^ However, the role of quinoa‐derived FA in antitumor and reversing tumor chemoresistance remains unclear.

DDX6 (Rck/p54) is a member of the DEAD‐box family of deconjugating enzymes, which is highly conserved from unicellular eukaryotes to vertebrates. DDX6 has a wide range of functions, including initiating translation, splicing mRNA, assembling ribosomes, and participating in RNA processing.^[^ ^17^ ^]^ It has been shown that DDX6 is overexpressed in most malignant cell lines and contributes positively to the development of various cancers, so inhibiting DDX6 expression or blocking its interactions with other proteins involved in tumor progression may be a reasonable approach to impede tumor progression.^[^ ^18^, ^19^ ^]^ DDX6 is abnormally elevated in human pancreatic cancer tissues and is crucial for supporting cell survival and proliferation.^[^ ^20^ ^]^ It also promotes the invasion of lung adenocarcinomas.^[^ ^21^ ^]^ These factors are important in the advancement of cancer and resistance to treatment. DDX6 also encourages resistance to radiochemotherapy in glioblastoma.^[^ ^22^ ^]^ In gastric cancer cells, DDX6 has been shown to interact with c‐Myc mRNA, leading to increased levels of c‐Myc mRNA and protein.^[^ ^23^ ^]^ Furthermore, DDX6 can facilitate colorectal cancer proliferation by activating the classical Wnt signaling pathway.^[^ ^24^ ^]^ However, there is a lack of research on how DDX6 regulates BC and its resistance. Therefore, developing drugs that target DDX6 and its associated signaling pathways is becoming a prominent focus in efforts to combat BC resistance.

In this study, we demonstrated that FA enhanced chemosensitivity by inhibiting ABC transporter proteins, thereby promoting the accumulation of chemotherapeutic agents in vivo. Using drug affinity response target stability (DARTS) and biotin pull‐down techniques, DDX6 was revealed to be the direct target protein of FA, and H378 was the key amino acid. FA enhanced the interactions between DDX6 and HSC70, leading to the degradation of DDX6 via CMA, which impaired GSK‐3β/β‐catenin signaling and reduced β‐catenin cellular accumulation, thereby combating resistance. Consequently, our findings suggest that DDX6 is a potential new target for the treatment of BC chemoresistance and paves the way for the development of FA as a novel chemosensitizing drug to overcome BC resistance.

## 2 Result

### 2.1 FA Reverses BC Chemoresistance in Vivo and in Vitro

**Figure 1:**
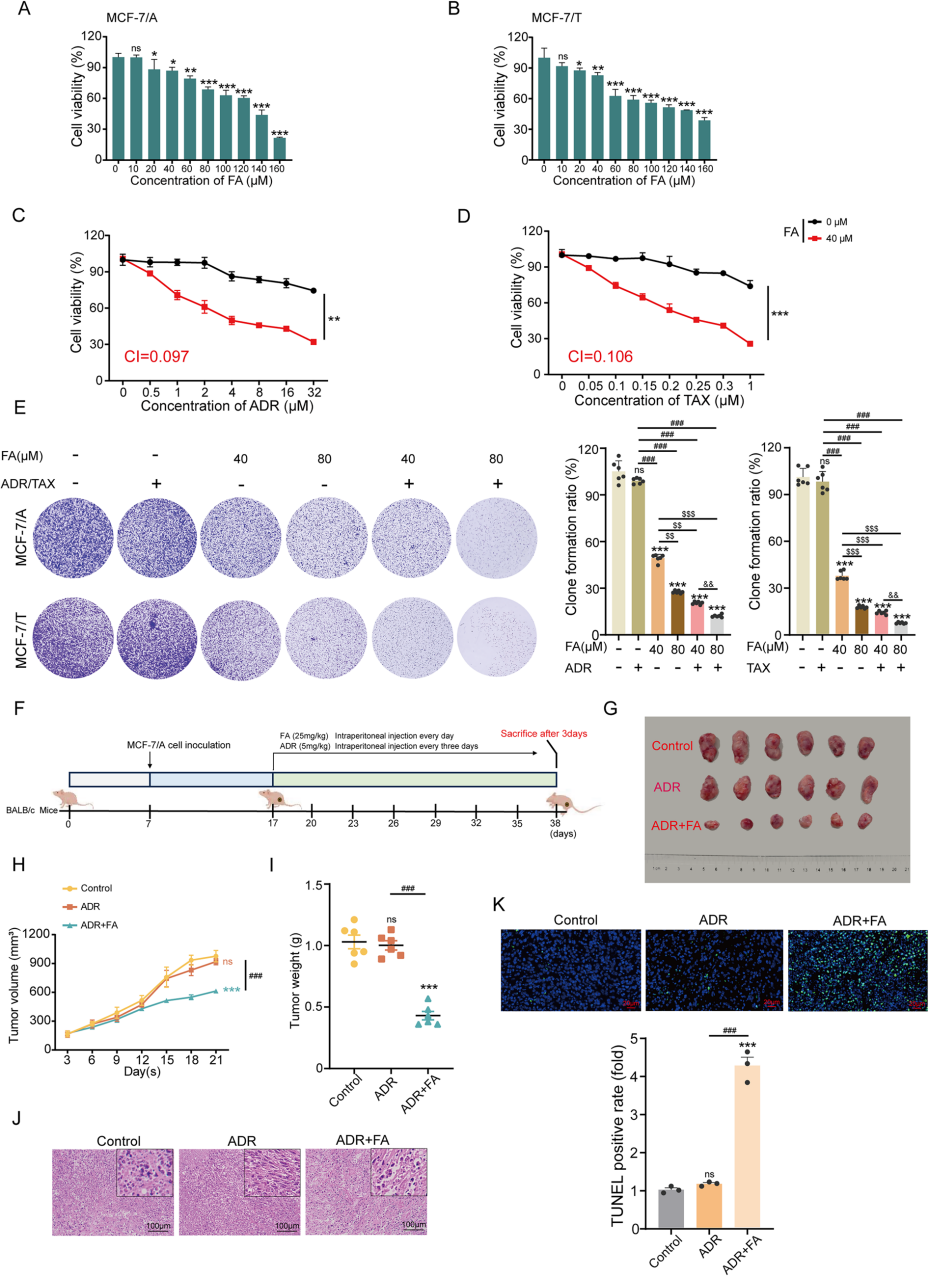
FA enhances the sensitivity of drug‐resistant cells to ADR/TAX. A,B) Cell viability of MCF‐7/A and MCF‐7/T cells was determined by Cell Counting Kit 8 (CCK8) after treating cells with different concentrations of FA for 48 h, n = 3. C,D) Growth inhibition curves of resistant cells treated with ADR and TAX alone or in combination with FA to determine the resistance reversal index (RI) and combination index (CI), n = 3. E) Resistant cells were exposed to chemotherapeutic drugs (ADR, TAX) and FA (40, 80 µm) alone or together for 48 h and analyzed by colony formation assay after 7 days (left panel). Relative colony formation rates are expressed as SEM ± mean (right panel), n = 6. F) Schematic diagram of the experimental design of the xenograft tumor mouse model. G) Representative images of tumors in mice subcutaneously inoculated with MCF‐7/A cells and treated with 5 mg kg^−1^ of ADR or in combination with 25 mg kg^−1^ of FA. H,I) Statistical analysis graphs of the volume and weight of the tumors, n = 6. J) H&E staining plots of tumors in different groups (scale bar, 100 µm), n = 3. K) Effect of ADR alone or combined treatment with FA on apoptosis of tumor tissues (scale bar, 20 µm), n = 3. Data are presented as mean ± SEM (A, B, C, D, E, H, I, and K) and were analyzed using Student's t‐test (C, and D) or one‐way ANOVA (A, B, E, H, and K) with Bonferroni's multiple comparisons test. *p < 0.05; **p < 0.01; ***p < 0.001 versus control. ###*p* < 0.001 versus ADR or TAX‐treated group. $$*p* < 0.01; $$$*p* < 0.001 versus 40 µm FA treatment. &&*p* < 0.01 versus 40 µm FA in combination with ADR/TAX. ns, not significant.

The drug resistance index is utilized to evaluate the resistance of cancer cells to chemotherapeutic agents. To confirm the resistance of MCF‐7/A and MCF‐7/T cells to ADR and TAX, the CCK8 assay was performed, and the resistance index was subsequently calculated. As shown in Figure S1A (Supporting Information), the IC_50_ values of MCF‐7/A and MCF‐7/T cells to ADR and TAX were significantly increased by 12.5‐fold and 10.6‐fold, respectively, compared with parental MCF‐7 cells (Table S1, Supporting Information). Moreover, the expression of ABC transporter proteins was also significantly increased versus parental MCF‐7 cells (Figure S1B, Supporting Information). Notably, the results in **Figure** **1**A,B indicated that FA exhibited significant inhibitory effects on MCF‐7/A and MCF‐7/T cells, where the IC_50_ values were 109.4 and 110.6 µm, respectively, and FA (0–140 µm) had almost no toxic effect on normal human mammary epithelial cells (MCF‐10A) (Figure S1C, Supporting Information). Treatment of cells with the chemotherapeutic drugs ADR / TAX alone or FA in combination, the results demonstrated that FA significantly enhanced the susceptibility of resistant cells to ADR and TAX, with a resistance reversal fold of 10.3 and 9.4. The combination index (CI) was below 0.8, strongly evidencing the synergistic effect of the combination of FA and ADR/TAX (Figure 1C,D and **Table** **1**). The combined doses of low/high concentration FA (40 / 80 µm) and ADR/TAX (32µm /1 µm) were selected for follow‐up experiments. In addition, the colony‐forming ability of resistant cells was more inhibited by the combination of FA and chemotherapeutic agents than by FA alone (Figure 1E). To further investigate the effect of FA on chemosensitivity in vivo, we constructed a xenograft tumor model by selecting a combination of FA and ADR that is more effective in inhibiting colony formation (Figure 1F). The results indicated that the use of ADR did not reverse the chemoresistance of BC, whereas the co‐treatment of FA reduced the tumor volume and weight and significantly overcame the drug resistance (Figure 1G–I). Meanwhile, hematoxylin and eosin (H&E) staining revealed that FA altered the morphology of the transplanted tumors (Figure 1J). TUNEL staining further proved that FA promoted apoptosis (Figure 1K). We also noticed no significant differences between the treatment and control groups regarding body weight and histological characteristics of key organs (Figure S1D,E, Supporting Information). These results suggest that FA could enhance the therapeutic sensitivity, and the combination of FA and ADR/TAX exerts synergistic inhibitory effects on MCF‐7/A and MCF‐7/T cells.

**Table 1:** FA increases chemosensitivity in drug‐resistant BC cells to ADR and TAX.

Chemotherapy drug	IC_50_ [µm]	Reversal fold
ADR	149±1.77	10.3
ADR+FA	14.4±3.98	
TAX	4.7±2.35	9.4
TAX+FA	0.5±0.29	

### 2.2 FA Enhances Chemosensitivity by Inhibiting ABC Transporter Proteins

**Figure 2:**
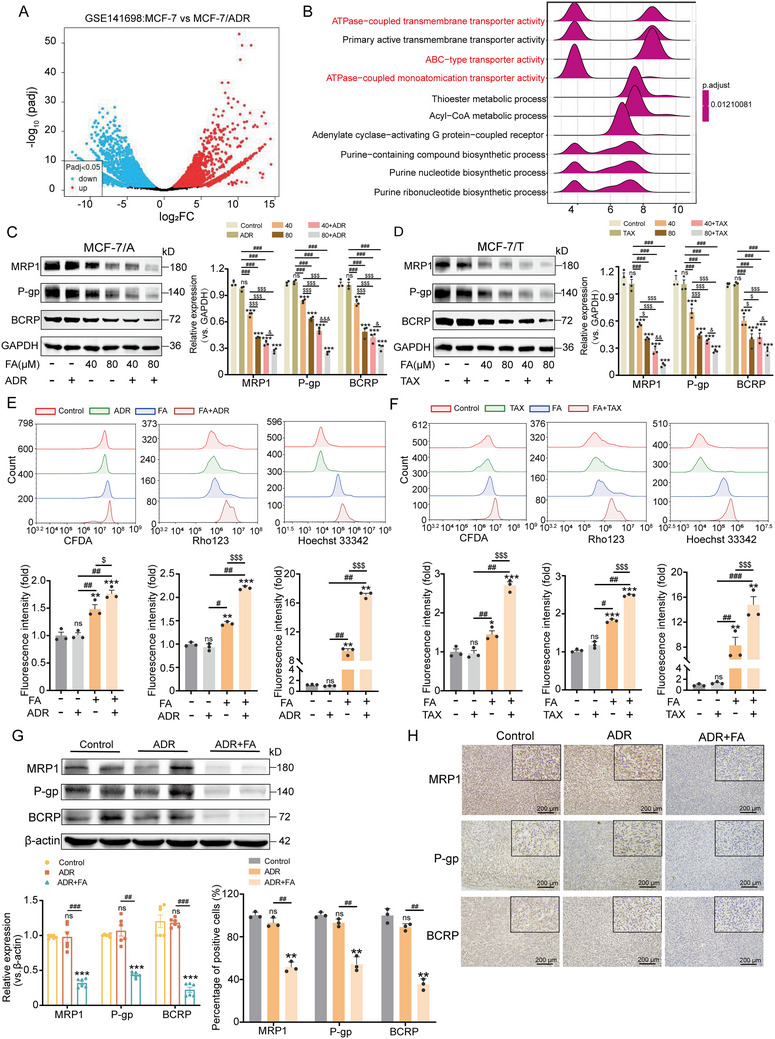
FA inhibits ABC transporter proteins to reduce drug efflux. A) Volcano plot showing differentially expressed genes between MCF‐7 and MCF‐7/A cells. B) GSEA analysis of differentially expressed genes in MCF‐7 and MCF‐7/A cells. C,D) Resistant cells treated with chemotherapeutic agents (ADR, 32 µm / TAX, 1 µm) and FA (40, 80 µm) alone or in combination for 48 h, immunoblotting to analyze the expression of MRP1, P‐gp, and BCRP, n = 3. E,F) Resistant cells treated with chemotherapeutic agents (ADR, 32 µm / TAX, 1 µm) and FA (80 µm) alone or in combination for 48 h, fluorescence intensities of CFDA, Rho123, and Hoechst 33342 were analyzed by flow cytometry (upper panel). Relative fluorescence intensities are expressed as SEM ± mean (bottom panel), n = 3. G) Immunoblotting analysis of MRP1, P‐gp, and BCRP in tumor tissues, n = 3. H) Representative images of IHC staining of MRP1, P‐gp, and BCRP in tumor tissues (scale bar, 200 µm), n = 3. Data are presented as mean ± SEM (C, D, E, F, G, and H) and were analyzed using one‐way ANOVA (C, D, E, F, G, and H) followed by Bonferroni's multiple comparisons test. *p < 0.05; **p < 0.01; ***p < 0.001 versus control. #*p* < 0.05; ##*p* < 0.01; ###*p* < 0.001 versus ADR or TAX‐treated group; $*p* < 0.05; $$$*p* < 0.001 versus 40 or 80 µm FA‐treated group. &*p* < 0.05; &&*p* < 0.01; &&&*p* < 0.001 versus 40 µm FA in combination with ADR/TAX. ns, not significant.

The results in Figure 1 indicate that FA inhibited chemoresistance in BC cells, but it is still unknown how FA reverses chemoresistance. RNA sequencing data (GSE141698) downloaded from the TCGA database, Gene set enrichment analysis (GSEA) showed that the triggering of ADR resistance was mainly due to enhanced activity of ATP‐binding cassette (ABC) transporter proteins compared to MCF‐7 cells (**Figure** **2**A,B), implying that ABC transporter proteins may be essential for BC drug resistance. We further investigated the effect of FA on the expression of ABC transporter proteins. The results of WB and qPCR revealed that FA significantly decreased the expression of MRP1, P‐gp, and BCRP, while the combination of FA with ADR/TAX produced a stronger inhibitory effect than FA alone (Figure 2C,D; Figure S2A, Supporting Information). Interestingly, the half‐lives of MRP1, P‐gp, and BCRP did not change significantly after FA treatment, suggesting that FA did not affect the stability of ABC transporter proteins (Figure S2B, Supporting Information). Because 5‐carboxyfluorescein diacetate (CFDA), Rhodamine 123 (Rho123), and Hoechst 33342 are classical substrates for MRP1, P‐gp, and BCRP, respectively, we subsequently assessed whether FA treatment alone or in combination with ADR/TAX altered CFDA, Rho123, and Hoechst 33342 uptake. The results indicated that the FA promoted the in vivo accumulation of ADR and TAX and reduced exocytosis, and the combined drug was more efficacious (Figure 2E,F). In addition, immunohistochemistry (IHC) and WB of tumor tissues suggested that the combination of FA and ADR significantly reduced the expression of the proteins MRP1, P‐gp, and BCRP (Figure 2G,H). To further validate the inhibitory effect of FA on ABC transporter proteins, we used MK‐571 (MRP1 inhibitor), Zosuquidar (P‐gp inhibitor), and Ko143 (BCRP inhibitor), which compared the effects of these inhibitors with those of FA or FA in combination with ADR/TAX. Notably, only Ko143 showed superior inhibition compared to FA, whereas the combination of FA with ADR/TAX produced the most pronounced reversal of resistance (Figure S3, Supporting Information). Collectively, these results demonstrate that FA reduces the expression of ABC transporter proteins, thereby contributing to the accumulation of chemotherapeutic agents and enhancing chemosensitivity.

### 2.3 FA Reduces β‐Catenin Accumulation in BC‐Resistant Cells

**Figure 3:**
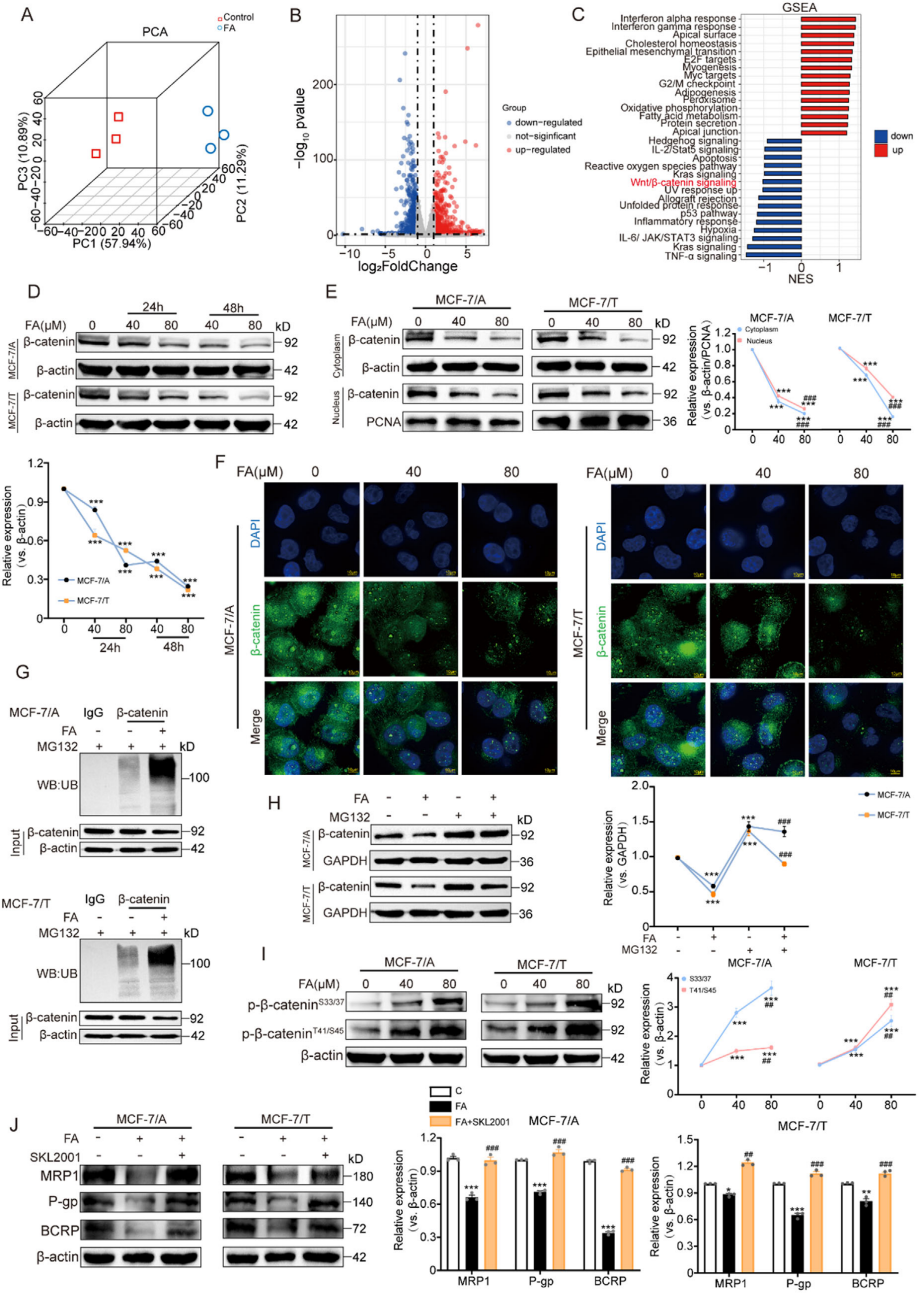
FA promotes phosphorylation of β‐catenin and targets β‐catenin for ubiquitin‐proteasome degradation. A) Principal component analysis of MCF‐7/A cells in control and FA‐treated groups, n = 3. B) Volcano diagram showing the differentially expressed genes in the control and FA‐treated groups. C) GSEA analysis of differentially expressed genes in control and FA‐treated groups. D) Immunoblotting analysis of β‐catenin protein after FA treatment at different times and concentrations, n = 3. E) Immunoblotting analysis of β‐catenin protein levels in the nucleus and cytoplasm of FA‐treated drug‐resistant cells, n = 3. F) Immunofluorescence analysis of nuclear and cytoplasmic fractions of β‐catenin. The green fluorescent signal represents the β‐catenin protein, and the blue fluorescence signal represents the nucleus (scale bar: 10 µm), n = 3. G) Detection of the effect of FA on ubiquitination of β‐catenin. Cell lysates treated with C+MG132 or FA+MG132 were immunoprecipitated (IP) with an anti‐β‐catenin antibody and immunoblotted (IB) with an anti‐ubiquitin antibody, n = 3. H) Immunoblotting analysis of β‐catenin protein levels in cells treated with FA or FA+MG132, n = 3. I) Assessment of β‐catenin protein levels phosphorylated on different amino acid residues, including serine (S) 33, 37, 45, and threonine (T) 41 in drug‐resistant cells, n = 3. J) Immunoblotting analysis of MRP1, P‐gp, and BCRP proteins by treatment with FA or FA+SKL2001, n = 3. Data are presented as mean ± SEM (D, E, H, I, and J) and were analyzed using one‐way ANOVA (D, E, H, I, and J) followed by Bonferroni's multiple comparisons test. *p < 0.05; **p < 0.01; ***p < 0.001 versus control. ##*p* < 0.01; ###*p* < 0.001 versus 40 or 80 µm FA‐treated group.

To elucidate the molecular mechanism of FA for reversing chemoresistance, we performed RNA sequencing on resistant cells treated with or without FA. The results showed that the FA‐treated and control groups were distinctly differentiated, with 1583 genes differentially expressed between the two groups, of which 882 genes were up‐regulated and 701 genes were down‐regulated by FA treatment (**Figure** **3**A,B). Among these downregulated genes were MRP1, P‐gp, and BCRP (Figure S4A, Supporting Information). Unexpectedly, GSEA analysis of these genes indicated that treatment with FA had a significant impact on the Wnt signaling pathway (Figure 3C). Furthermore, an analysis of the Kyoto Encyclopedia of Genes and Genomes (KEGG) of the dataset that caused resistance (GSE141698) suggested significant enrichment within this same pathway as well (Figure S4B,C, Supporting Information). Therefore, β‐catenin, the central hub of the Wnt signaling cascade, was further focused. We observed that β‐catenin was downregulated by FA within 24 h, and the downregulation was more pronounced with increasing time and concentration (Figure 3D). Next, we found that FA significantly inhibited β‐catenin cytoplasmic and nuclear accumulation, which is a key feature of Wnt/β‐catenin signaling activation and transcription of drug‐resistant genes (Figure 3E,F). In tumor tissues, we consistently detected an inhibitory effect of FA on β‐catenin levels (Figure S4D, Supporting Information). These findings further support that FA inhibits β‐catenin to combat chemoresistance.

In contrast to its inhibitory effect on β‐catenin, FA did not affect the mRNA level of β‐catenin (CTNNB1) in BC‐resistant cells (Figure S4E, Supporting Information). We hypothesized that FA‐mediated down‐regulation of β‐catenin could be due to protein degradation. To verify this hypothesis, we employed the proteasome inhibitor MG132 to block the ubiquitin‐proteasome system. The results showed that FA treatment resulted in increased ubiquitination of β‐catenin protein (Figure 3G). Consistently, the reduction in β‐catenin expression induced by FA was reversed upon addition of MG132 (Figure 3H), suggesting that FA reduces the expression of β‐catenin by enhancing its protein hydrolysis. It is well known that ubiquitination protein hydrolysis of β‐catenin is tightly controlled by the phosphorylation status of specific sites, for example, phosphorylation of S33/37/45 and T41 sites enhances the degradation of β‐catenin.^[^ ^25^ ^]^ Consistent with these reports, we found that FA significantly increased phosphorylation levels at S33/37/45/T41 sites on β‐catenin, thereby promoting its degradation (Figure 3I). These findings imply that FA facilitates ubiquitinated protein hydrolysis of β‐catenin by phosphorylating β‐catenin. The accumulation of β‐catenin in the cytoplasm and nucleus was significantly reduced, as was the expression level of target genes such as ABC transporter proteins, which are activated by the binding of β‐catenin to transcription factors in the nucleus. We next used SKL2001, a known β‐catenin activator, and the anti‐resistance effect of FA was weakened with the addition of SKL2001 (Figure 3J). These observations reveal that the anti‐resistance activity of FA is mainly dependent on the blockade of the β‐catenin signaling pathway.

### 2.4 DDX6 Identified as a Direct Target of Effect for FA

**Figure 4:**
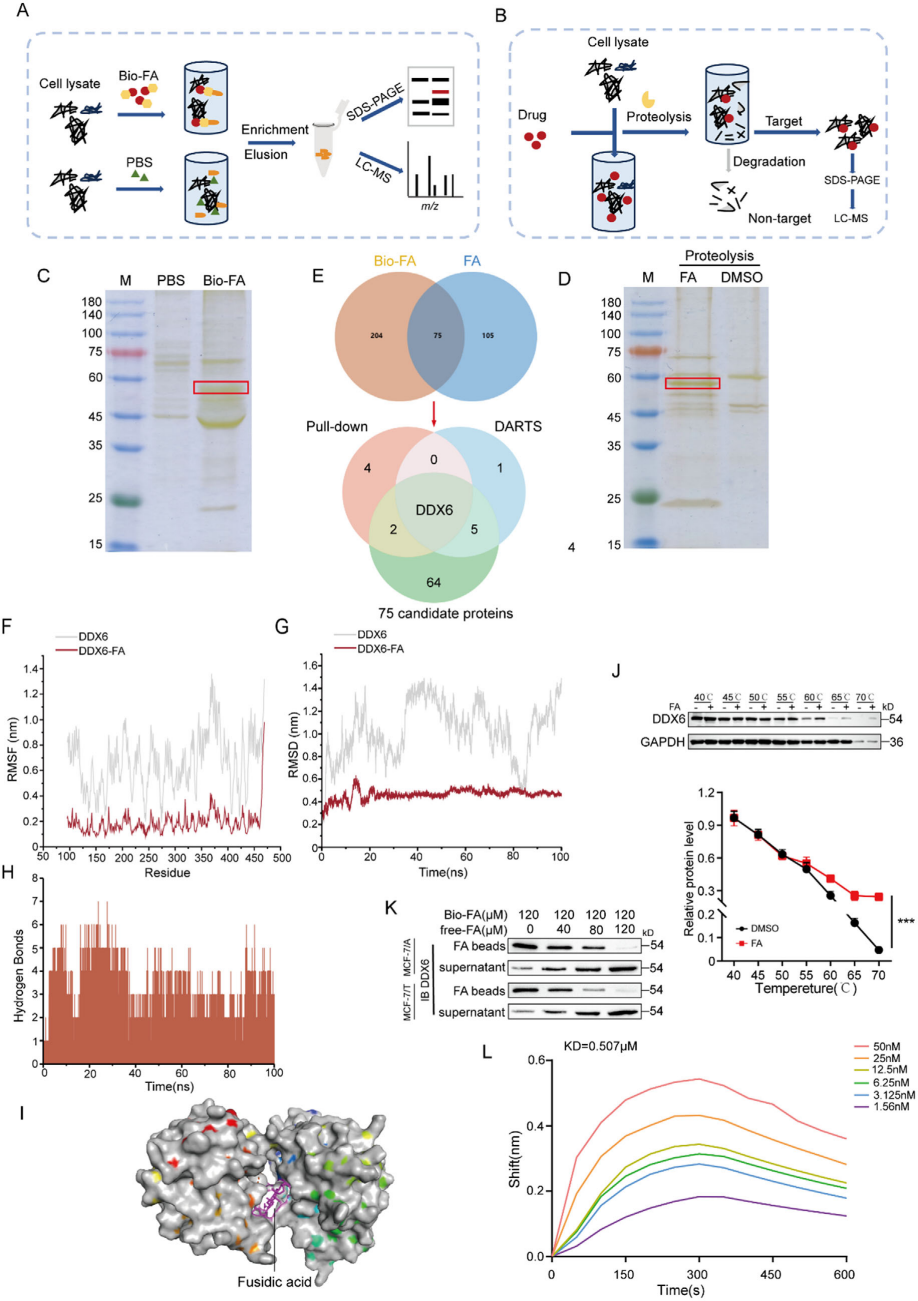
FA binds directly to the target protein DDX6. A) Flowchart of pull‐down assay using biotin‐FA. B) Flowchart of using DARTS to obtain stable binding of the target protein to FA. C) Schematic diagram of silver staining for pull‐down assay using biotin‐FA. D) Schematic diagram of silver staining using DARTS to obtain target proteins stably bound to FA. E) Analysis of the interaction of target proteins by biotin pull‐down with DARTS acquired using Venn Diagrams. F) RMSF is calculated based on MD simulation trajectories. G) Time‐dependent changes in the RMSD of the DDX6 and FA‐DDX6 complex during MD simulation. H) Changes in the number of hydrogen bonds between FA and DDX6 during MD simulation. I) The binding energy of FA to DDX6 was analyzed by molecular docking. J) Immunoblotting analysis of CETSA of MCF‐7/A cell lysates incubated with FA or an equal volume of vector at the indicated temperatures (upper panel). The band density of DDX6 was obtained from Western blot results (lower panel), n = 3. K) Co‐incubation with free‐FA and bio‐FA to detect DDX6 expression under competitive binding, n = 3. L) His‐DDX6 was purified in vitro, and the equilibrium association constant (KD) of FA binding to DDX6 was analyzed using Gator. Data are presented as mean ± SEM (J) and were analyzed using Student's t‐test. ***p < 0.001 versus the DMSO group.

To gain insight into the pharmacological mechanism of FA blocking the β‐catenin signaling pathway, we used the pull‐down and DARTS strategy to dissect the target proteins of FA (**Figure** **4**A–D). The mass spectrometry analysis revealed 75 candidate target proteins for FA. We subsequently cross‐referenced these candidates with the top ten proteins identified in the MaxQuant scoring from both DARTS and pull‐down experiments, leading to the identification of four genes associated with drug resistance: DDX6, DSP, ANXA2, and HRNR. Following this, we conducted interference experiments on these four genes and found that only DDX6 inhibited the expression of MRP1, P‐gp, and BCRP (Figure 4E; Figure S5A–C, Supporting Information). To further assess the stability of FA binding to DDX6, we performed molecular dynamics (MD) simulations. Specifically, we extracted conformations of the complex at time points 0, 10, 30, 50, 70, 90, and 100 ns. Throughout the simulations, FA remained stably anchored within its binding pocket, indicating a sustained interaction with DDX6 (Figure S6A, Supporting Information). Additionally, root mean square fluctuation (RMSF) analysis, which reflects the flexibility of the protein, showed that the RMSF value for the FA‐DDX6 complex was lower than for DDX6 alone (Figure 4F). Similarly, root mean square deviation (RMSD) analysis, which measures the overall movement of the complex, revealed that the value of RMSD was reduced for the FA‐DDX6 complex compared to DDX6 alone (Figure 4G). Furthermore, the radius of gyration (Rg), which describes the degree of compactness of the protein mimicry structure, exhibited lower values in the FA‐DDX6 complex (Figure S6B, Supporting Information). Hydrogen bonding, a key noncovalent interaction, was monitored throughout the 100 ns simulation, and the FA‐DDX6 complexes had 0 to 7 hydrogen bonds, usually around 3, which highlights the role of hydrogen bonding in maintaining the stable binding of FA to DDX6 (Figure 4H). Molecular docking simulations also revealed that the binding energy of FA to DDX6 was −8.5 kcal (Figure 4I). The results of the CETSA assay indicated that FA significantly enhanced the thermal stability of DDX6 compared to the control (DMSO‐treated), even at higher temperatures, implying that FA binds to DDX6 (Figure 4J). Furthermore, we observed that DDX6 was significantly pulled down by Bio‐FA beads in competitive binding assays, whereas excess FA effectively blocked the binding of DDX6 to Bio‐FA beads (Figure 4K). Subsequently, we demonstrated the specific interaction between DDX6 and FA using purified DDX6 protein to assess the binding capacity of FA through surface plasmon resonance (SPR) analysis. The data showed that FA bound to DDX6 with an equilibrium association constant (KD) of 0.507 µm (Figure 4L), suggesting that FA has a moderate affinity for DDX6, further supporting that DDX6 is a target of FA.

### 2.5 FA Depends on DDX6 to Regulate the GSK‐3β/β‐Catenin Axis for its Anti‐Resistance Activity

**Figure 5:**
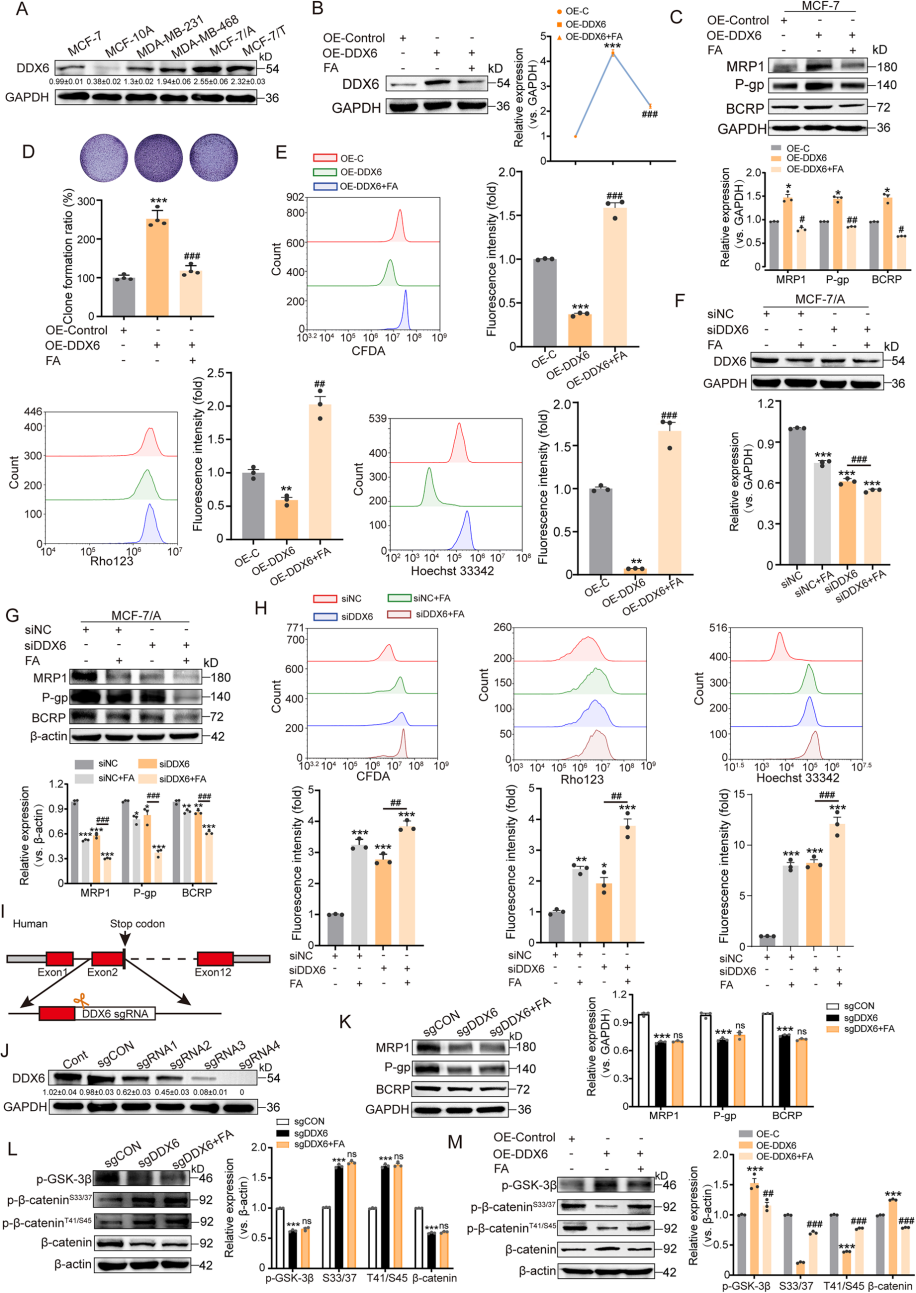
DDX6 is essential for chemoresistance in BC. A) Expression of DDX6 in BC MCF‐7, MDA‐MB‐231, MDA‐MB‐468, MCF‐7/A, MCF‐7/T cells, and normal mammary epithelial MCF‐10A cells was analyzed by immunoblotting, n = 3. B) MCF‐7 cells were transfected with pCMV‐DDX6‐HA, and cells were collected 48 h later to detect DDX6 expression, n = 3. C) Overexpression of DDX6 and treatment with FA (80 µm) in MCF‐7 cells to detect the effect on the expression of drug‐resistant proteins by immunoblotting, n = 3. D) MCF‐7 cells were transfected with pCMV‐DDX6 and treated with FA (80 µm) for 48 h at the same time, and the colony formation of the cells was analyzed (upper panel). Relative colony formation rates are expressed as SEM ± mean (bottom panel), n = 4. E) Effect of DDX6 overexpression and FA intervention on CFDA, Rho123, and Hoechst 33342 fluorescence intensity, n = 3. F) MCF‐7/A cells were transfected with siDDX6, and DDX6 expression was analyzed by immunoblotting after DDX6 knockdown and FA (80 µm) treatment, n = 3. G) Knockdown of DDX6 and treatment with FA (80 µm) in MCF‐7 cells to detect the effect on the expression of drug‐resistant proteins by immunoblotting, n = 3. H) Effect of DDX6 knockdown and FA intervention on CFDA, Rho123, and Hoechst 33342 fluorescence intensity, n = 3. I) Experimental strategy to establish DDX6 knockout cell lines by using CRISPR/Cas9. J) Immunoblotting analysis of DDX6 knockout efficiency after different sgRNA transfection, n = 3. K) Effects of knockout of DDX6 and FA intervention on the levels of different drug‐resistant proteins, n = 3. L) Effects of knockout of DDX6 and FA intervention on phosphorylated GSK‐3β and β‐catenin, n = 3. M) Effect of DDX6 overexpression and FA intervention on phosphorylated GSK‐3β and β‐catenin, n = 3. Data are presented as mean ± SEM (B, C, D, E, F, G, H, K, L, and M) and were analyzed using one‐way ANOVA (B, C, D, E, F, G, H, K, L, and M) followed by Bonferroni's multiple comparisons test. *p < 0.05; **p < 0.01; ***p < 0.001 versus control. #*p* < 0.05; ##*p* < 0.01; ###*p* < 0.001 versus overexpression of DDX6 or knockdown of DDX6. ns, not significant.

It is crucial to understand whether the target protein DDX6 is beneficial for the inhibitory effect of FA on chemoresistance. Our analysis utilizing the GEPIA database (https://gepia.cancer‐pku.cn) revealed that DDX6 was expressed at significantly higher levels in BRCA tissues compared to normal tissues, and that high expression of DDX6 was associated with poor prognosis (Figure S7A–C, Supporting Information). Protein blotting analysis was further performed to evaluate the protein levels of DDX6 in drug‐resistant cells (MCF‐7/A, MCF‐7/T) and their parental cells (MCF‐7), as well as other breast cancer cells (MDA‐MB‐231 and MDA‐MB‐468) and normal human breast cells (MCF‐10A). The results indicated that DDX6 protein expression was significantly elevated in cancer cells compared to normal cells. Notably, drug‐resistant cells exhibited higher levels of DDX6 than their parental cells. We further selected MCF‐7 and MCF‐7/A for subsequent experiments, as they were identified as cells with lower and higher DDX6 expression, respectively (**Figure** **5**A). In addition, western blot analysis found that MRP1, P‐gp, and BCRP expression increased upon DDX6 overexpression (Figure 5B,C). As shown in Figure 5D,E, overexpression of DDX6 significantly enhanced the colony‐forming ability of cells and promoted the efflux of CFDA, Rho123, and Hoechst 33342, whereas the opposite phenomenon was observed in siRNA knockdown cells (Figure 5F–H; Figure S7D, Supporting Information). FA treatment reversed or enhanced the phenotypic changes associated with resistance induced by DDX6 overexpression or knockdown (Figure 5B–H). To further determine whether the reversal of resistance by FA was DDX6‐dependent, we knocked out DDX6 in MCF‐7/A cells using CRISPR/Cas9. The results indicated that the knockout of DDX6 significantly diminished the expression of MRP1, P‐gp, and BCRP, whereas the inhibitory effect of FA was blocked, suggesting that FA targets DDX6 to exert its anti‐resistance effect (Figure 5I–K).

It is known that β‐catenin degradation is dependent on reduced phosphorylation of glycogen synthase kinase‐3β (GSK‐3β) and that the reduction of p‐GSK‐3β triggers β‐catenin hydrolysis by phosphorylating β‐catenin at the S33/37/ 45/T41 sites.^[^ ^26^ ^]^ Therefore, we hypothesized that DDX6 regulates chemoresistance by modulating the p‐GSK‐3β/β‐catenin axis. Consistently, our findings demonstrated that FA and DDX6 knockout decreased p‐GSK‐3β (S9) but increased p‐β‐catenin (S33/37/45/T41), leading to β‐catenin degradation (Figure 5L). In contrast, overexpression of DDX6 initiated p‐GSK‐3β signaling, inhibited p‐β‐catenin (S33/37/45/T41), and upregulated β‐catenin (Figure 5M). Similarly. In the patient's tumor tissue, DDX6 expression was positively correlated with MRP1, P‐gp, MRP1, and β‐catenin (Figure S7E, Supporting Information). Overall, these results support that FA exerts its effect against chemoresistance by targeting DDX6 expression to disrupt the GSK‐3β/β‐catenin axis.

### 2.6 FA‐Promoted Enhancement of DDX6 and HSC70 Interactions Drives DDX6 Protein Degradation via CMA

**Figure 6:**
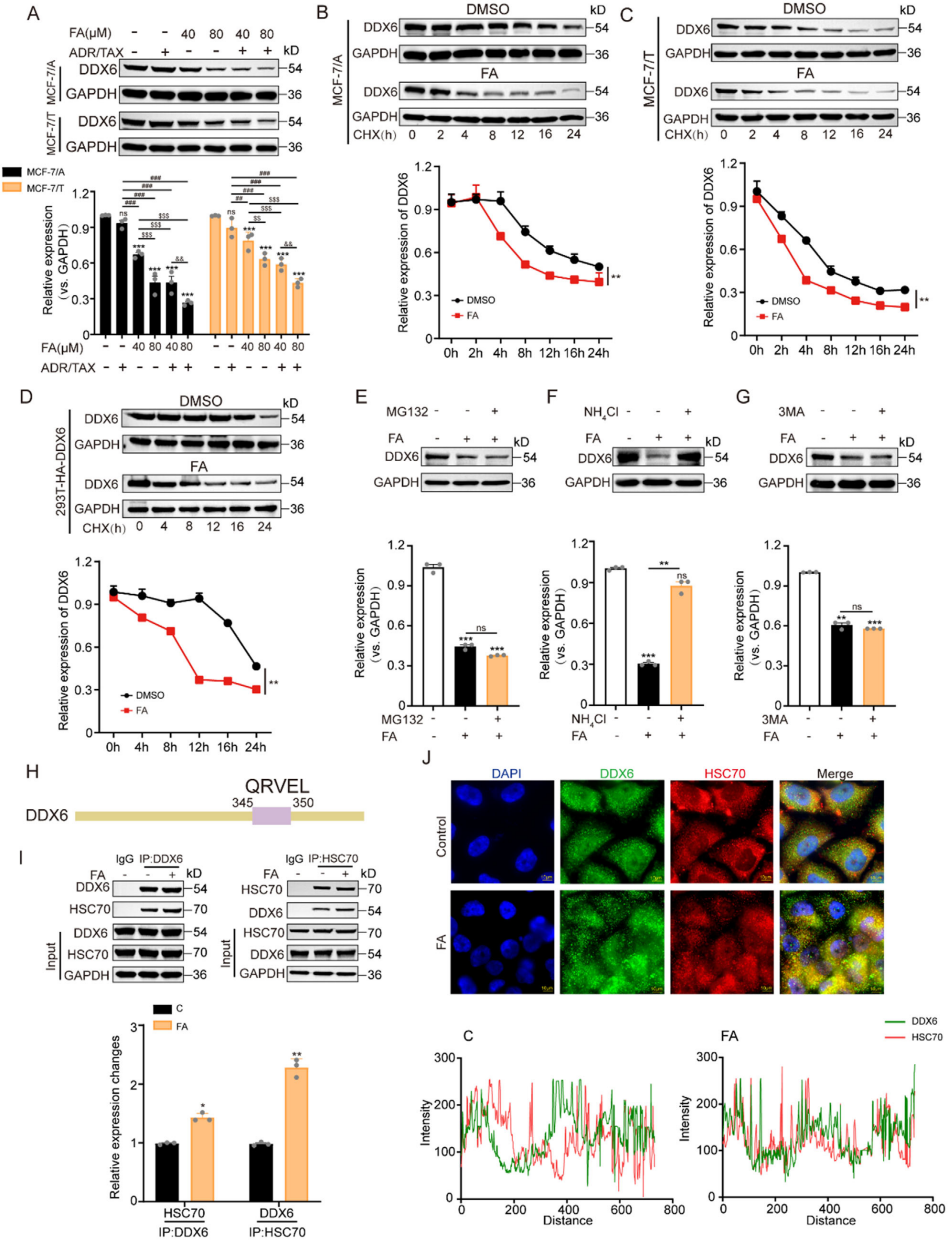
FA promotes DDX6 degradation through CMA. A) Resistant cells were treated with chemotherapeutic agents (ADR, 32 µm / TAX, 1 µm) and FA (40, 80 µm) alone or in combination for 48 h. The expression of DDX6 was analyzed by immunoblotting, n = 3. B,C) MCF‐7/A (B) and MCF‐7/T cells C) were treated with DMSO (control) and FA, and the half‐life of DDX6 was determined by the CHX Chase assay. The quantitative results are shown below, n = 3. D) After transfecting pCMV‐DDX6‐HA in 293T cells, the half‐life of exogenous DDX6 in control and FA‐treated groups was determined by the CHX‐chase assay. The quantitative results are shown below, n = 3. E–G) Resistant cells were treated with 20 µm MG132 E), 25 mm NH_4_Cl F), or 5 mm 3MA G) alone or co‐treated with 80 µm FA, and the expression level of DDX6 was detected by immunoblotting, n = 3. H) Schematic representation of the KEFRQ motif of DDX6. I) The effects of FA treatment on DDX6 and HSC70 interactions were determined by CO‐IP and western blot, n = 3. J) Immunofluorescence analysis of the co‐localization of DDX6 and HSC70 in cells after FA treatment (scale bar, 10 µm), n = 3. Data are presented as mean ± SEM (A, B, C, D, E, F, G, and I) and were analyzed using Student's t‐test (B, C, D, and I) or one‐way ANOVA (A, E, F, and G) with Bonferroni's multiple comparisons test. *p < 0.05; **p < 0.01; ***p < 0.001 versus DMSO or control or 80 µm FA treatment. ##*p* < 0.01; ###*p* < 0.001 versus ADR or TAX‐treated group; $$*p* < 0.01; $$$*p* < 0.001 versus 40 µm FA‐treated group. &&*p* < 0.01 versus 40 µm FA in combination with ADR/TAX. ns, not significant.

Given the significant role of DDX6 in BC resistance, we explored the potential inhibitory mechanism of FA on DDX6 protein expression. We observed that DDX6 protein expression was significantly decreased after treatment with FA, ADR, and TAX alone. Notably, co‐treatment with FA and ADR/TAX exhibited more potent inhibition of DDX6 expression compared to either treatment alone, implying that FA enhanced the sensitivity of chemotherapeutic agents in the cells by reducing DDX6 expression (**Figure** **6**A). Subsequently, we examined the half‐life of DDX6 in drug‐resistant cells following treatment with cycloheximide (CHX), a protein synthesis inhibitor, and it was shown that FA promotes the degradation of DDX6 (Figure 6B,C). Consistently, FA treatment significantly shortened the half‐life of exogenous HA‐DDX6 in 293 T cells (Figure 6D). To elucidate the pathways involved in FA‐mediated down‐regulation of DDX6, we treated cells with inhibitors targeting various pathways. Our findings revealed that supplementation with the lysosomal inhibitor NH_4_Cl, but not the proteasomal inhibitor MG132, rescued the reduced DDX6 expression due to FA intervention, suggesting that lysosomal inhibitors are involved in FA‐mediated DDX6 degradation (Figure 6E,F). Next, the mechanism of FA‐mediated lysosome‐dependent degradation of DDX6 was investigated. As shown in Figure 6G, inhibition of autophagy initiation by the autophagosome formation inhibitor 3‐Methyladenine (3‐MA) did not impede FA‐induced lysosomal degradation of DDX6, which indicates that other degradation pathways are involved in this process. Molecular chaperone‐mediated autophagy (CMA) represents one such lysosome‐mediated protein degradation pathway. It is characterized by its selectivity and specificity toward target proteins when compared to microautophagy or macroautophagy. All CMA substrates must contain at least one KFERQ‐like motif that can be recognized by HSC70.^[^ ^27^ ^]^ Sequence analysis indicated that DDX6 contains a putative KFERQ‐like motif (Figure 6H). We subsequently assessed the binding affinity between DDX6 and HSC70. Immunoprecipitation assays revealed an interaction between DDX6 and HSC70 that was enhanced by FA treatment (Figure 6I,J). In conclusion, our results suggest a novel mechanism whereby FA promotes DDX6 degradation via CMA.

### 2.7 His378 of DDX6 is Critical for Binding to FA

**Figure 7:**
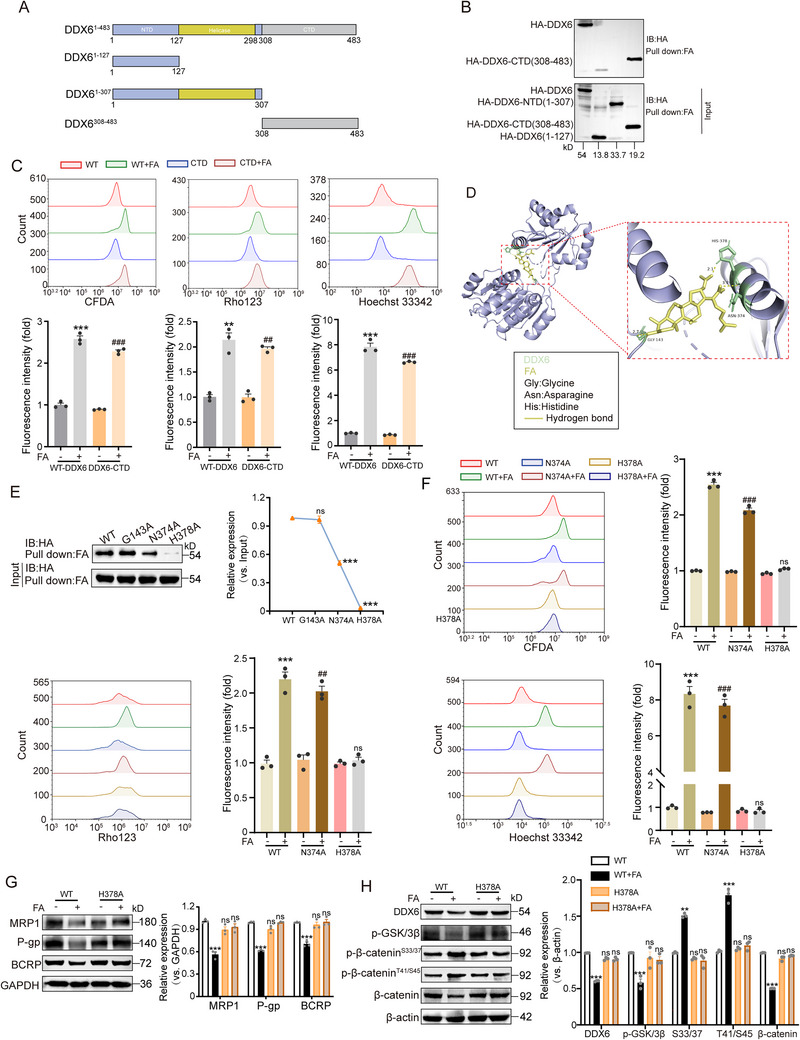
FA increases chemosensitivity by directly targeting the H378 residue of DDX6. A) Schematic representation of human DDX6 truncation mutants. NTD, N‐terminal regulatory domain; Helicase, Helicase domain; CTD, C‐terminal regulatory domain. B) The 293T cells were transfected with pCMV‐HA‐DDX6, pCMV‐HA‐DDX6‐NTD (1‐307aa), pCMV‐HA‐DDX6 (1‐127aa), and pCMV‐HA‐DDX6‐CTD (308‐483aa). The cells were collected after 48 h and evaluated by pull‐down assay for the binding of the three DDX6 truncated mutants to FA, n = 3. C) MCF‐7 cells were transfected with pCMV‐HA‐DDX6 and pCMV‐HA‐DDX6‐CTD and treated with 80 µm FA for 48 h. The effects of wild‐type and CTD‐truncated mutants of DDX6 on the fluorescence intensities of CFDA, Rho123, and Hoechst 33342 were measured by flow cytometry, n = 3. D) Binding patterns of FA to DDX6 based on molecular modeling experiments. The detailed view shows that FA (yellow) forms hydrogen bonds with key amino acid residues (yellow dashed line). E) Targeting mutant DDX6 binds G143, N374, and H378 in the pocket, the 293T cells were transfected with wild‐type DDX6 or 3‐site mutants, and binding of the three mutants of DDX6 to FA was evaluated by pull‐down assay, n = 3. F) The pCMV‐HA‐DDX6, pCMV‐HA‐DDX6^N374A^_,_ and pCMV‐HA‐DDX6^H378A^ were transfected into MCF‐7 cells and treated with 80 µm FA for 48 h. The effects of DDX6 wild‐type, N373A, and H378A on CFDA, Rho123, and Hoechst 33342 fluorescence intensity were detected by flow cytometry, n = 3. G) Expression of drug‐resistant proteins in wild‐type or H378 mutant cells treated with FA was determined by immunoblotting, n = 3. H) Expression of DDX6, p‐GSK‐3β, β‐catenin, and p‐β‐catenin proteins in wild‐type or H378 mutants treated with FA was determined by immunoblotting, n = 3. Data are presented as mean ± SEM (C, E, F, G, and H) and were analyzed using one‐way ANOVA (C, E, F, G, and H) followed by Bonferroni's multiple comparisons test. **p < 0.01; ***p < 0.001 versus transfected wild‐type DDX6. ##*p* < 0.01; ###*p* < 0.001versus transfected CTD truncation mutant or site mutants. ns, not significant.

DDX6 consists of 483 amino acids and contains a deconvolution enzyme motif, N‐terminal structural domain, and C‐terminal structural domain. To determine the region of action of FA on DDX6, the construct of DDX6 was truncated into three segments: NTD domain (1‐307aa), helicase domain (1‐127aa), and CTD domain (308‐483aa) (**Figure** **7**A). Pull‐down assays utilizing wild‐type or truncated forms of DDX6 demonstrated that FA binds effectively to the CTD structural domain (Figure 7B). PI‐Annexin V staining and drug efflux experiments indicated that the effect of FA on the CTD domain was comparable to the wild‐type DDX6. Thus, the CTD structural domain was identified as the region of FA interaction with DDX6 (Figure 7C; Figure S8A, Supporting Information). Next, we used molecular docking to investigate the amino acids that bind DDX6 to FA. The docking results revealed that FA establishes favorable hydrogen bonding interactions with residues such as Gly143, Asn374, and His378 (Figure 7D). To pinpoint the most critical residues for FA binding, we mutated several of these interacting residues and found that N374A and H378A showed reduced binding to FA and both amino acids were located in the CTD domain, which is consistent with the results described above, so we speculated that N374 and H378 might be the amino acids that FA interacts with DDX6 (Figure 7E). Subsequent PI‐Annexin V staining and drug efflux experiments revealed that the H378 mutant affected FA‐induced accumulation of CFDA, Rhodamine123, and Hoechst33342 and apoptosis, whereas the A374 mutation did not prevent the anti‐resistance effect of FA, which suggests that H378 is a key amino acid in the interaction of FA with DDX6 (Figure 7F; Figure S8B, Supporting Information). MD simulations also proved that FA did not stably anchor in the binding pocket after 50 ns throughout the simulations, indicating that the H378 mutation affects the interaction of FA with DDX6. The H378 mutation increased the values of the RMSD, RMSF, and Rg of the complexes, causing the stability to be reduced, and the binding energy to be changed from ‐33.98 kcal in the wild type to ‐21.6 kcal (Figure S8C–F, Supporting Information). We further examined the effect of the H378 mutation on the GSK‐3β/β‐catenin axis. The results showed that the H378 mutation completely halted the effect of FA, and no sustained down‐regulation of drug‐resistant proteins, β‐catenin, and p‐GSK‐3β protein was observed, nor was there any further elevation of p‐β‐catenin levels (Figure 7G,H). These findings suggest that residue H378 is situated within a critical hydrophobic pocket of DDX6‐CTD and plays a direct role in FA binding.

### 2.8 In Vivo Validation of FA to Overcome Chemoresistance via DDX6

**Figure 8:**
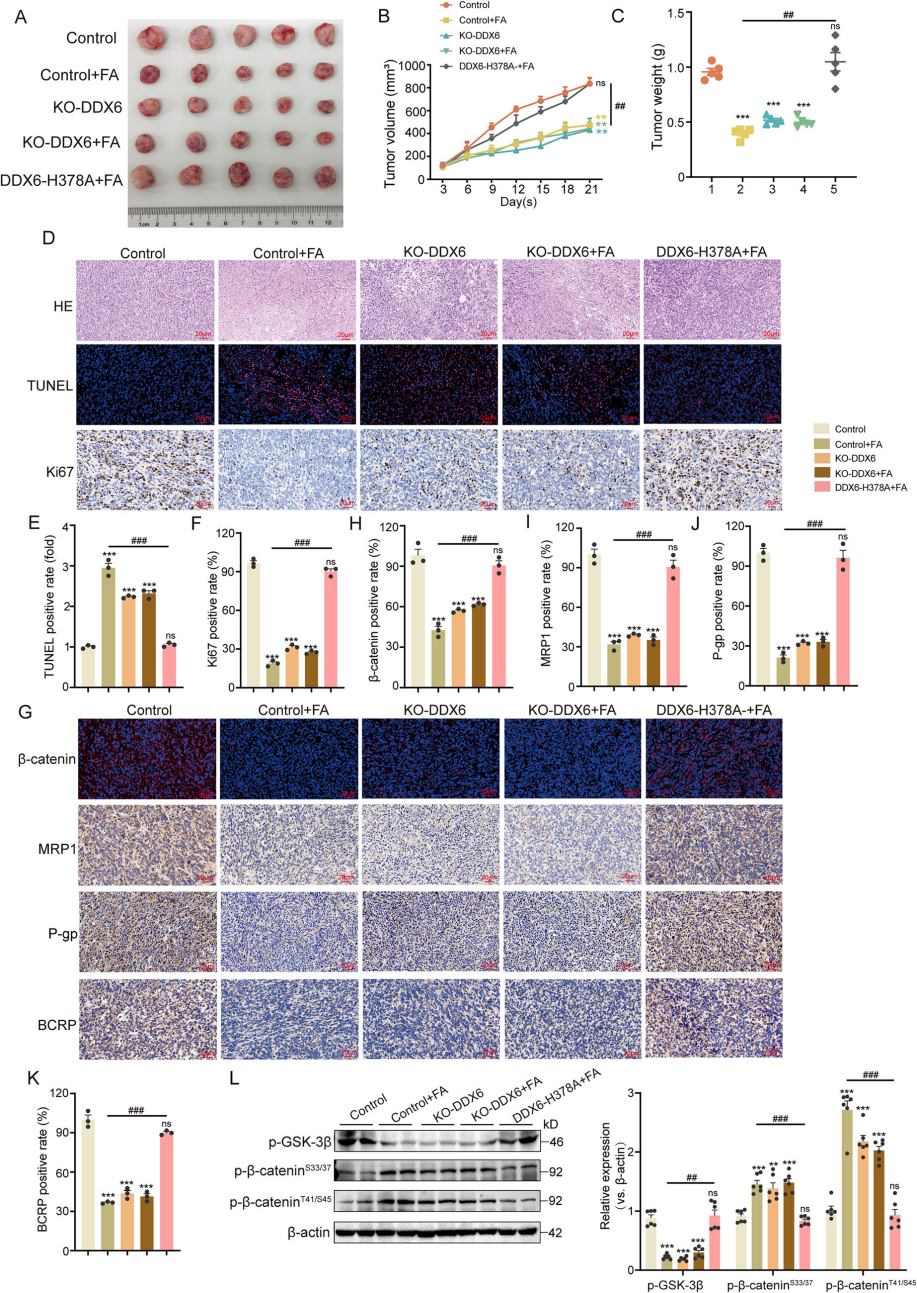
FA inhibits xenograft tumor growth by targeting DDX6 and H378 residues. A) MCF‐7/A cells with stable knockdown of DDX6, cell lines expressing DDX6‐H378A, were subcutaneously injected into nude mice to establish a xenograft model, and the FA treatment group was injected with FA (25 mg kg^−1^) or DMSO daily to show representative images of tumor growth for different treatments, n = 5. B) Graphs showing the tumor growth curves of different treatments, n = 5. C) Graphs showing tumor weights of different treatments, n = 5. D) Representative images of H&E staining, TUNEL staining, and Ki67 IHC staining of tumors between different groups (scale bar, 20 µm), n = 3. E,F) Statistical analysis of the positive rates of TUNEL staining and Ki67 staining in tumor tissues, n = 3. G) Representative pictures of immunofluorescence staining for β‐catenin, and IHC staining for MRP1, P‐gp, and BCRP in tumors between different groups (scale bar, 20 µm), n = 3. H–K) Statistical analysis of the positive rates of β‐catenin, MRP1, P‐gp, and BCRP staining in tumor tissues, n = 3. L) Western blotting to detect the expression of phosphorylated GSK‐3β and β‐catenin in tumors, n = 3. Data are presented as mean ± SEM (B, C, E, F, H, I, J, K, and L) and were analyzed by Student's t‐test (B) or one‐way ANOVA (C, E, F, H, I, J, K, and L) with Bonferroni's multiple comparisons test. **p < 0.01; ***p < 0.001 versus control group. ##*p* < 0.01; ###*p* < 0.001 versus FA treatment group. ns, not significant.

We further verified the importance of DDX6 for chemotherapy resistance in vivo. Stably expressing MCF‐7/A cells with knockout (KO) for DDX6 and those harboring the DDX6‐H378A were subcutaneously transplanted into Balb/c nude mice and subsequently treated intraperitoneally with FA. Tumor images (**Figure** **8**A) and statistical analyses (Figure 8B,C) revealed that FA treatment and knockout of DDX6 resulted in reduced tumor size and weight. However, FA was ineffective following the knockout of DDX6. The inhibitory effect of FA was also hindered after introducing the H378 mutation. Further histological analysis using H&E staining showed disorganized tumor cells exhibiting morphological irregularities in FA therapy or following KO‐DDX6. Conversely, no therapeutic effect was observed within either KO‐DDX6 or DDX6‐H378A groups when treated with FA. Consistently, TUNEL staining showed that administration of FA alongside knockout of DDX6 promoted apoptosis, whereas no apoptosis was seen in the KO‐DDX6 or DDX6‐H378A groups, in contrast to the expression of the proliferation marker Ki67 (Figure 8D–F). Similar to the above results, the expression of drug‐resistant proteins MRP1, P‐gp, BCRP, and β‐catenin decreased following FA intervention, whereas no significant changes were observed in the DDX6 knockout and mutant groups after FA treatment (Figure 8G–K). Finally, we examined the expression of some phosphorylated proteins in tumor tissues and obtained results that aligned with our in vitro experiments (Figure 8L). Taken together, these data indicate that DDX6 is a potential therapeutic target for BC resistance, and FA exerts anti‐drug resistance by inhibiting DDX6.

## 3 Discussion

FA is a commonly used clinical antibiotic for antibacterial and anti‐inflammatory therapy.^[^ ^28^, ^29^, ^30^, ^31^ ^]^ Our previous research indicated that terpenoid QBT extracted from quinoa possesses anti‐tumor activity as well as drug‐resistance reversal effects.^[^ ^16^ ^]^ However, the pharmacological activity and underlying mechanisms of FA, its main component, have yet to be thoroughly investigated. In the present study, we found that an important cause of BC resistance is the overexpression of ABC transporter proteins that mediate the efflux of drugs and are particularly known as ABCB1, ABCG2, and ABCC1, indicating that the inhibition of these transporter proteins has been developed as a possible mechanism to combat the effects of resistance.^[^ ^10^ ^]^ Fan et al. found that Dacomitinib, a small‐molecule tyrosine kinase inhibitor (TKI), antagonized multidrug resistance in cancer cells by inhibiting the efflux activity of ABCB1 and ABCG2 transporter proteins.^[^ ^32^ ^]^ Additionally, Zhang et al. found that the macrolide dilactone compound FW‐04‐806 significantly enhanced the cytotoxicity of chemotherapeutic agents on ABCB1 or ABCG2 overexpressing cells both in vitro and in vivo, suggesting its role in reversing drug resistance.^[^ ^33^ ^]^ Herein, we provide evidence that FA also exhibits a defeating effect on chemoresistance induced by ABC transporter proteins. This was attributed to its inhibition of MRP1, P‐gp, and BCRP, which effectively blocked drug efflux. Consistently, FA showed significant efficacy in reversing resistance in xenograft tumor models. These findings strongly suggest that FA has the potential to be a clinical drug to overcome BC resistance.

To reveal the signaling pathways of FA effects, we performed GSEA analysis on the RNA sequencing data and KEGG analysis on the dataset causing ADR resistance in BC. The Wnt/β‐catenin pathway caught our attention. Because Wnt/β‐catenin was the only intersection signaling pathway in the two analyses, comprehensive experimental validation was performed to ensure that FA's anti‐resistance activity was attributable to its effect on the Wnt/β‐catenin pathway. The Wnt/β‐catenin pathway plays a critical role in tumor progression and significantly influences cancer‐related mortality. It also impedes cell apoptosis and mediates drug resistance.^[^ ^34^, ^35^, ^36^ ^]^ The key player in Wnt is β‐catenin, and this pathway is balanced by regulating the cytoplasmic level of β‐catenin.^[^ ^37^ ^]^ When β‐catenin accumulates at high levels in the cytoplasm, it migrates to the nucleus to affect the expression level of genes involved in biological mechanisms. Therefore, interfering with Wnt/β‐catenin signaling may be an effective strategy to overcome chemoresistance.^[^ ^38^, ^39^ ^]^ In this context, the identification of FA as a novel inhibitor of the Wnt pathway that controls β‐catenin degradation is of great importance. Our findings indicate that FA inhibits GSK‐3β phosphorylation, which contributes to the phosphorylation of β‐catenin residues S33, S37, S45, and T41, leading to their ubiquitination and proteasomal degradation. In particular, FA reversal resistance was attenuated when SKL2001, an agonist for the Wnt/β‐catenin signaling pathway, stabilized intracellular β‐catenin by disrupting Axin/β‐catenin interactions. Moreover, given that Wnt/β‐catenin is intricately linked with self‐renewal and maintenance of stemness in BC stem cells, further investigation into FA's regulatory role concerning stemness warrants attention.^[^ ^40^, ^41^ ^]^

The identification and validation of bioactive molecular targets remain a critical step in the field of chemical biology and drug discovery. Affinity‐based pull‐down and DARTS are reliable techniques for drug target protein identification.^[^ ^42^, ^43^ ^]^ In this study, DDX6 was identified as a direct target of FA. DDX6 is an RNA‐binding protein that acts as an oncogene in a variety of cancers and promotes tumor growth by activating the classical Wnt pathway.^[^ ^24^ ^]^ However, the functional and clinical significance of DDX6 in BC remains unknown. The current study revealed that DDX6 is upregulated in cancer tissue and predicts poor prognosis in BC patients. More importantly, through gain and loss of function studies, DDX6 significantly regulates BC resistance. Furthermore, based on the results of MD and pull‐down experiments, we demonstrate that the CTD domain and H378 residue of DDX6 are essential for FA binding to DDX6 as well as its anti‐resistance properties. Interestingly, DDX6 has been shown to promote the maintenance of cell stemness, providing strong evidence that FA regulates stemness reversal resistance.^[^ ^44^ ^]^

These results indicate that FA treatment inhibits the expression of DDX6 in MCF‐7/A and MCF‐7/T cells by promoting the CMA. CMA is a lysosomal degradation pathway that eliminates substrate proteins through HSC70 protein recognition, translocation assisted by lysosome‐associated membrane protein type 2A, and degradation of target proteins containing KFERQ motifs, which makes CMA highly selective among the three autophagy pathways.^[^ ^45^, ^46^ ^]^ To investigate whether lysosomal involvement is necessary for the degradation of DDX6 protein, we first administered the lysosomal inhibitor NH_4_Cl along with the proteasome inhibitor MG132. Our observations revealed that treatment with NH_4_Cl reversed the reduction of DDX6 due to FA intervention, suggesting that lysosomes are involved in FA‐induced downregulation of DDX6 in BC‐resistant cells. We further found that the addition of 3‐MA, an inhibitor of autophagy initiation, did not prevent FA‐mediated lysosomal degradation of DDX6. This indicates that additional degradation pathways may be involved in this process. Importantly, the analysis revealed that DDX6 contains a KFERQ motif, which is a potential substrate for CMA. Here, we further showed that DDX6 interacts with HSC70 and noted an enhancement in this interaction following FA treatment, further demonstrating that FA treatment accelerated the degradation of the DDX6 protein in these BC‐resistant cells. These findings provide experimental evidence that FA inhibits DDX6 protein expression via the CMA pathway.

Our findings indicate that FA is effective in overcoming BC resistance in vitro and in vivo by inhibiting ABC transporter proteins. Based on the TCGA database and functional experiments, DDX6 has proved to be critical in inducing BC resistance. FA directly binds to residue H378 of DDX6, enhances its interaction with HSC70, leads to the degradation of the CMA of DDX6, which decreases the downstream level of p‐GSK‐3β, leading to the upregulation of p‐β‐catenin (S33/37/45/T41). It then facilitates the hydrolysis of β‐catenin proteins via the ubiquitin‐proteasome pathway, thereby decreasing the expression levels of MRP1, P‐gp, and BCRP, and rendering cells more susceptible to chemotherapeutic agents. These results highlight that FA is a promising natural compound that warrants further investigation as a chemotherapeutic adjuvant to combat BC resistance.

## 4 Experimental Section

### Chemicals and Antibodies

FA was from Aladdin (Shanghai, China). Adriamycin (ADR), taxol (TAX), cycloheximide (CHX), 5‐carboxyfluorescein diacetate (CFDA), rhodamine 123 (Rho123), and Hoechst 33342 were from Solarbio (Beijing, China). The antibodies against MRP1, P‐gp, BCRP, β/catenin, DDX6, Ubiquitin, HA, and Ki67 were purchased from Proteintech (Wuhan, China). The antibodies against HSC70 were purchased from Bioss (Beijing, China). The p‐β/catenin and p‐GSK‐3β antibodies were from Cell Signaling Technology (Shanghai, China). Ammonium chloride (NH_4_Cl), MG‐132, 3‐Methyladenine (3‐MA), Zosuquidar, MK571 and Ko143 were purchased from MCE (Shanghai, China).

### Cell Culture

Breast cancer adriamycin‐resistant cells (MCF‐7/ADR) and breast cancer taxol‐resistant cells (MCF‐7/TAX) were purchased from Wing and Applied Biotechnology Co. (Shanghai, China). MCF‐7/A and MCF‐7/T cell lines were cultured in 1640 medium containing 10% fetal bovine serum. To maintain drug resistance, 500 ng mL^−1^ ADR was added to the medium for culturing MCF‐7/A cell lines, and 100 ng mL^−1^ TAX was added to MCF‐7/T cell lines. HEK‐293T cells were cultured in DMEM medium containing 10% fetal bovine serum. All cell lines were cultured at 37 °C in a humid incubator with 5% CO_2_.

### CCK8 Assay

For the drug resistance analysis experiment, the BC‐resistant cells with good growth status and their parental cells MCF‐7 were taken and inoculated into 96‐well plates, 100 µL of cell suspension was added to each well, and blank wells were set up at the same time (no cells were added, but the same volume of medium was added), after the cells adhered to the wall, different concentrations of ADR/TAX were added to treat them respectively, and at the end of the treatment, 10 µL of CCK8 was added to each well, and then the cells were continued to be incubated for 1–4 h. Subsequently, the absorbance at 450 nm of the cells was measured with an enzyme labeler. Calculation of resistance index (RI).

(1) $$\begin{array}{cc} Resistance\;Index\left(RI\right) & =Half\;inhibitory\;concentration\left(IC_{50}\right)\\ & \;of\;chemotherapeutic\;drugs\;by\;resistant\;cells/IC_{50}\\ & \;of\;chemotherapeutic\;drugs\;by\;parental\;cells \end{array}$$

To perform the resistance reversal assay, different concentrations of FA or ADR and TAX alone or FA in combination with ADR and TAX were treated after BC‐resistant cells adhered to the plate, and the resistance reversal fold (RF) was calculated at the end of the treatment.

(2) $$\begin{array}{cc} Reversal\;Fold\left(RF\right) & =IC_{50}\;of\;resistant\;cells\;to\;chemotherapeutic\;agents\\ & \;alone/IC_{50}\;of\;resistant\;cells\;to\;chemotherapeutic\\ & \;agents\;in\;combination\;with\;FA \end{array}$$

To calculate the coadministration index (CI), since the action concentration of FA with a cell viability greater than 80% was chosen, its contribution was ignored in the CI formula, and only the dose ratio of the chemotherapeutic agent was calculated, and determined synergistic effects if the CI < 1.

(3) $$\begin{array}{cc} \mathrm{Comb}\mathrm{inat}\mathrm{ion}\;\mathrm{Index}\left(\mathrm{CI}\right) & =IC_{50}\;\mathrm{of}\;\mathrm{the}\;\mathrm{chem}\mathrm{othe}\mathrm{rape}\mathrm{utic}\;\mathrm{agent}\;\mathrm{in}\\ & \;\mathrm{comb}\mathrm{inat}\mathrm{ion}\;\mathrm{with}\;\mathrm{FA}/IC_{50}\;\mathrm{of}\;\mathrm{the}\\ & \;\mathrm{chem}\mathrm{othe}\mathrm{rape}\mathrm{utic}\;\mathrm{agent}\;\mathrm{alone} \end{array}$$

### Clone Formation Experiment

Take the drug‐resistant cells in the logarithmic growth phase, blow them into single cells by trypsin digestion, suspend them in a complete medium, inoculate the cell suspension into 6‐well plates at a density of 5000 cells/well for further cultivation, and carry out the treatment of drug or transfection, and then change the medium after 48 h, and thereafter change the medium every 3 days to observe the state of the cells. After clone formation, the cells were fixed with 4% paraformaldehyde for 30 min, then rinsed with PBS, then stained with crystal violet staining solution for 20 min, then rinsed with PBS several times, and then air‐dried and photographed. After recording, the crystal violet was dissolved with 1% SDS, and the OD value was measured at 570 nm to count the change in cell proliferation ability.

### Western Blot Assay

Cells from different treatments were collected, and total protein was extracted and quantified by BCA. Protein samples were mixed with SDS‐PAGE sample buffer and denatured by heating. The samples were loaded into the gel wells and separated by electrophoresis. The electrophoresed gel was transferred to a PVDF membrane. After membrane transfer, the membrane was sealed with the protein‐free sealing solution for 20 min at room temperature to reduce the non‐specific binding sites on the membrane. The membranes were incubated overnight at 4 °C with the corresponding diluted primary antibody. The next day, the membrane was washed with TBST to remove the unbound primary antibody, followed by incubation of the corresponding secondary antibody at room temperature for 2 h. The membrane was washed again with TBST to remove the unbound secondary antibody, and the changes in protein expression on the membrane were observed using the chemiluminescent solution.

### Drug Efflux Assay

Drug‐resistant MCF‐7/A and MCF‐7/T cells were treated with ADR/TAX and FA alone or in combination for 48 h. Subsequently, the cells were exposed to 1 µm Rho 123 (substrate for P‐gp), 2.5 µm Hoechst 33342 (classical substrate for BCRP), and 1 µm CFDA (MRP1‐specific substrate) for 20 min, and the staining solution was washed out with PBS to remove excess staining solution. After centrifugation, the extra staining solution was washed away with PBS, and the fluorescence intensity was detected by flow cytometry.

For the inhibition of ABC transporter protein, the resistant cells were treated with 80 µm FA, 1 µm zosuquidar (P‐gp inhibitor), 10 µm MK571 (MRP1 inhibitor), and 1 µm Ko143 (BCRP inhibitor) for 48 h. The cells were collected, stained, and analyzed by flow cytometry.

To determine the role of DDX6 on drug resistance and the structural domains and sites where FA interacts with DDX6, siDDX6 was transfected into MCF‐7/A cells, and pCMV‐DDX6‐HA and its truncates and mutants were transfected into MCF‐7 cells, and the cells were similarly treated with FA for 48 h. The cells were collected, stained, and analyzed by flow cytometry.

### Immunohistochemistry (IHC)

Tumor tissue samples were fixed in 4% paraformaldehyde, and the fixed tissues were paraffin‐embedded and cut into thin slices. The slices were dewaxed and hydrated in xylene and ethanol. Subsequently, antigen repair was performed using EDTA buffer. Serum was used to close the non‐specific binding sites after repair. The diluted primary antibody was applied to the sections and incubated. Further signal enhancement was done using a secondary antibody, and the final color was developed using enzyme‐labeled substrates. After dehydration and transparency treatment, the sections were capped with dendrimer, and the experimental results were observed under the microscope.

### RNA Sequencing

Total RNA was isolated from MCF‐7/A cells using Trizol (Beijing, China) with or without FA incubation for 48 h. RNA quality was assessed, reverse transcribed to cDNA, the resulting cDNA library was sequenced, and the clean reads were quickly and accurately compared with the reference genome using Hisat2 (http://ccb.jhu.edu/software/hisat2) software to obtain information on the localization of the Reads on the reference genome. Mapped reads for each sample were assembled in a reference‐based approach using StringTie v1.3.1. For each transcribed region, FPKM (fragments per kb of transcript per million mapped reads) values were calculated using RSEM software to quantify their expression abundance and variation. Differential expression analysis of RNAs between two different groups (and between two samples by edgeR) was performed by DESeq2 software. Genes with a false discovery rate (FDR) parameter of less than 0.05 and absolute fold change ≥1 were considered differentially expressed.

### Immunofluorescence Assay

Drug‐resistant cells were inoculated in 24‐well plates containing round glass coverslips and treated with FA. After treatment, the cells were washed and fixed with pre‐cooled 4% paraformaldehyde. For mouse tumor tissues, tumors were fixed in 4% paraformaldehyde overnight, then dehydrated in 30% sucrose at 4 °C for 8 h. Tumors were then embedded in paraffin and cut into 6 µm‐thick slices. Cells or tumor tissues were permeabilized and closed, and then incubated with antibodies to the corresponding proteins at 4 °C overnight. The slides were stained with fluorescently labeled secondary antibody for 1 h at room temperature, and the nuclear cells were stained with DAPI. Fluorescence changes were visualized using fluorescence microscopy.

### Ubiquitination Assay

Resistant MCF‐7/A and MCF‐7/T cells were collected after treatment with MG132 (20 µm) alone or co‐treated with FA (80 µm), washed twice with pre‐cooled PBS, pre‐cooled lysis buffer was added to the cells, and cells were centrifuged at 13 000 g for 15 min at 4 °C, then the supernatant was transferred to a new centrifuge tube, which was then used as the total protein extract. Protein A/G agarose beads are prepared and rinsed twice with PBS. Antibodies (target and control) are pre‐coupled to the protein A/G agarose beads, and the total protein extract is incubated with the antibody carrier complex at 4 °C overnight with slow shaking. The next day, the beads were centrifuged, and the supernatant was discarded. The beads were washed three times with pre‐cooled PBS to remove non‐specifically bound proteins from the beads, and then the precipitated proteins were separated from the agarose beads and analyzed by Western blotting using anti‐ubiquitin antibody (Proteintech, 1:1000).

### Biotin Pull‐Down

Biotin‐labeled FA was conjugated to streptavidin‐functionalized agarose beads and incubated overnight with drug‐resistant cell protein lysate.FA and interacting proteins were immobilized on streptavidin agarose beads by biotin‐FA, and the non‐interacting proteins were washed away with PBS. The biotin‐FA‐protein complexes were subjected to SDS‐PAGE electrophoresis and silver staining to identify the differential bands, which were cut off and analyzed by MS.

### Drug Affinity Responsive Target Stability (DARST)

The MCF‐7/A cells were collected, and the total protein was extracted and divided into two equal portions. One portion was added to FA, and the other portion was added to DMSO, and incubated for 2 h at room temperature to allow FA to interact with the protein. Proteinase K was added at a final concentration of 1% (expressed as a percentage of protease mass to total protein), mixed well and incubated at 25 °C for 10 min, then 5 × SDS‐PAGE protein sampling buffer was added and boiled for 10 min, followed by SDS‐PAGE electrophoresis to isolate the processed protein samples and compare the degradation of the proteins in the presence of the drug with that in the absence of the drug. Silver staining was performed to intercept protein bands with altered stability upon binding to the drug, and identified by MS.

### MS Data Analysis

The MS data were analyzed using MaxQuant 2.0.1.0 to obtain information containing protein quantification, protein composition, etc., and the accuracy of protein identification was further assessed by parameters such as protein coverage, peptide number, and match quality. Finally, the top‐ranked proteins in terms of sequence coverage, protein score, and intensity‐based Absolute Quantification (iBAQ) were selected for validation analysis.

### Competitive Binding Assay

The drug‐resistant cells in good condition were collected and lysed to obtain protein supernatants, which were incubated with different concentrations of free‐FA (0, 40, 80, and 120 µm) for 4 h, and then incubated overnight with streptavidin‐affinity magnetic beads conjugated with Bio‐FA. The next day, the supernatant was collected, and the unbound proteins on the beads were rinsed with pre‐cooled PBS, mixed with the uploading buffer, boiled, and detected as changes in the corresponding proteins by SDS‐PAGE.

### CETSA‐WB

MCF‐7/A cells were lysed with RIPA lysis buffer, and then the cell lysates were incubated with DMSO or FA for 4 h. The cells were divided into 7 groups, boiled at different temperatures (40, 45, 50, 55, 60, 65, 70 °C) for 3 min, cooled at room temperature for 3 min, after centrifugation at 13 000 rpm for 15 min, the supernatant was added into loading buffer and the protein bands were detected by western blotting using an anti‐DDX6 antibody (Proteintech, 1:1000).

### Molecular Docking (MD)

Download the 3D structure of FA in SDF format from PubChem data, import the structure into ChemBio3D Ultra 14.0 for energy minimization, import the optimized small molecule into AutodockTools‐1.5.6 for hydrogenation, charge calculation, charge assignment, and set the rotatable key, and then save it as “ pdbqt ”format. DDX6 (PDB ID: 5ANR) was downloaded from the PDB database. Protein water of crystallization, primitive ligands, etc., were removed using Pymol 2.3.0, the protein structure was imported into AutoDockTools for hydrogenation, docking analysis was performed using AutoDock Vina1.1.2, and the docking results were visualized using Pymol 2.3.0.

### Molecular Dynamics (MD)

The crystal structure 5ANR of protein DDX6 was structurally repaired using Modeller v10.4 to construct the structure of wild‐type protein DDX6 and its complex with the small molecule Fusidic Acid. 100 ns MD simulations were performed using GROMACS 2020.6 software. Parameter and topology files of proteins and small‐molecule ligands were generated using a CHARMM36m force field. Periodic boundary conditions were set, and the dimensions of the simulated limiting box were optimized, whereby the proteins were placed in the center of the cubic box, and the distance to the edge of the box was at least 1.0 nm to fill the box with water molecules. To make the simulated system electrically neutral, some of the solvent water molecules were replaced with Na^+^ and Cl^−^ at a concentration of 0.15 mol L^−1^. The steepest descent method was used to minimize the energy consumption of the whole system, and the solvent and ions around the proteins were pre‐equilibrated to obtain a stable thermodynamic system under the desired conditions. The frog hopping algorithm was used for equilibrium kinetic integrals. All MD simulations were carried out at isothermal and isobaric pressure at a temperature of 303.15 K and a pressure of 1 bar for a duration of 100 ns.

### Gator® Molecular Interaction

Surface plasmon resonance (SPR) was measured using Gator Bio. The recombinant DDX6 protein was immobilized on the Anti‐His probe, and the immobilized sensor was subsequently immersed in samples containing different concentrations of FA to monitor the molecular binding process in real‐time, and to calculate the biological reaction parameters by analyzing the changes in reflectance spectra on the surface of the fiber‐optic biosensor. Finally, the binding data were analyzed using the software provided with the Gator system to calculate key parameters such as binding constants (kon), dissociation constants (koff), and equilibrium association constant (KD).

### CO‐IP

Drug‐resistant treated cells were collected and washed twice with pre‐cooled PBS. Pre‐cooled lysis buffer was added to the cells, and the cells were centrifuged at 13 000 g for 15 min at 4 °C. Then, the supernatant was transferred to a new centrifuge tube, and the supernatant was the total protein extract. Protein A/G agarose beads were prepared and washed twice with PBS. Antibodies (target and control) were pre‐coupled to the protein A/G agarose beads, and the total protein extract was incubated with the antibody carrier complexes, shaking slowly at 4 °C overnight. The next day, the beads were centrifuged and the supernatant discarded, rinsed three times with pre‐cooled PBS to remove non‐specific binding proteins from the beads, and then the precipitated proteins were separated from the agarose beads and analyzed by western blotting.

### Protein Stability Assay

The FA or DMSO‐treated resistant cells and 293T cells transfected with pCMV‐HA‐DDX6 for 48 h were treated with 60 µg mL^−1^ CHX for different times, the cells were collected, and protein expression changes were further detected by western blotting. In all experiments, the control group was prepared with the vector DMSO used for FA preparation, and the pixel values of the bands were calculated using ImageJ software to calculate the percentage of remaining proteins. The line graphs were created using GraphPad Prism software to analyze the protein half‐life.

### PE‐Annexin V Detection of Apoptosis

The treated drug‐resistant cells were collected according to the manufacturer's protocol, and the cells were resuspended and counted after PBS washing. Take 1–5 × 10^5^ resuspended cells, centrifuge and discard the supernatant, add 100 µL of diluted 1×Annexin V binding buffer to resuspend the cells, add 2.5 µL of Annexin V FITC reagent and 2.5 µL of PI reagent, gently vortexed and mixed, and then incubated at room temperature and protected from light for 15 min. Then 400 µL of 1 × Annexin V binding buffer was added, mixed, and immediately detected by flow cytometry.

### Plasmid Transfection, Targeted Mutation

siDDX6, siDSP, siANXA2, siHRNR, and siNC were manufactured by Gemma Genetics (Shanghai, China), and plasmids pCMV‐HA‐DDX6, truncated and mutant of DDX6, were synthesized by General Biosynthesis (Anhui, China). Specifically, the cells were cultured in 6‐well plates, and the medium was changed when the cell density reached 60%–70%, then transfected with Lipofectamine 3000, and the cells were collected 24–72 h later for subsequent experimental analysis.

### CRISPR/Cas9‐Mediated Gene Knockout

For gene knockout using CRISPR/Cas9 technology, annealed sgRNA oligonucleotides were cloned into the pX330 vector to generate the DDX6 knockout plasmid. After transfection, sgRNA knockout of DDX6 was screened, DDX6 gene‐deficient cells were selected with puromycin, and DDX6 gene defects were confirmed by western blot.

### Xenograft Tumor Model

All BALB/c‐Nude mice (male, 4 weeks old) were purchased from the Model Animal Research Center of Nanjing University and housed in specific pathogen‐free (SPF) cages in the Experimental Animal Center and Laboratory of the Department of Nephrology, Shanxi Provincial People's Hospital. The 5 × 10^6^ cell suspensions were injected subcutaneously into the flanks of the mice. When the tumor diameter reached 100 mm^3^, the mice were randomly divided into a drug administration group and a control group. 5 mg kg^−1^ ADR was administered intraperitoneally every 3 days in the ADR administration group, while 25 mg kg^−1^ FA was administered intraperitoneally daily in the FA group. Tumor size and body weight of the mice were measured every 3 days, and the tumor volume = (length × width^2^) / 2. Finally, tumors, hearts, livers, spleens, lungs, and kidneys were collected for western blot and histological analysis.

For the experiment to validate the target DDX6, mice were randomly divided into five groups, two of which were injected with MCF‐7/A cells, two were injected with the KO‐DDX6 cell line, and one group was injected with cells stably expressing DDX6^H378A^, and when the diameter of the tumors reached 100 mm^3^, the mice were treated with either intraperitoneal injection of 5 mg kg^−1^ ADR (every 3 days, Control and FA‐treated groups) or intraperitoneal administration of 25 mg kg^−1^ FA per day (three groups). After 3 weeks of treatment, the mice were euthanized, and the tumors were removed, weighed, and photographed. All animal experimental protocols were approved and accepted by the Laboratory Animal Ethics Committee of Shanxi University (Protocol Code: SXULL2023043).

### Statistical Analysis

Statistical analysis was performed using SPSS 27.0. All experiments were conducted as 3 independent experiments, and data are expressed as mean ± SEM. Sample sizes for each experiment and statistical test were provided in the legend. When the assumptions of normality and variance were met, comparisons between two groups were made using the two‐tailed Student's t‐test. For comparisons between three or more groups, one‐way analysis of variance (ANOVA) was performed, followed by the Bonferroni test to correct for multiple comparisons. Protein bands and average fluorescence intensity were quantified in grayscale using Image J. For all tests, *p* < 0.05 was considered statistically significant.

## Conflict of Interest

The authors declare no conflict of interest.

## Author Contributions

X.F. was responsible for the main experiments and data analysis, and wrote the MS; S.L. and J.T. performed the RNA sequencing analysis; X.F., D.G., and C.Z. performed the mouse experiments; Z.L. provided the conceptual ideas and expenses, and approved the MS.

## Supporting information



Supporting Information

## Data Availability

The data that support the findings of this study are available from the corresponding author upon reasonable request.
