# Supplementary material for: Fusidic Acid Reverses Chemoresistance in Breast Cancer via Targeting DDX6 to Downregulate GSK‐3β/β‐Catenin Signaling
Source: Adv Sci (Weinh). 2025 Aug 4;12(39):e04680. doi: 10.1002/advs.202504680 (PMC12981028; doi:10.1002/advs.202504680)
Supplement: Supplementary file 1 — Supporting Information [file ADVS-12-e04680-s001.docx]

**Supporting Information for the Original Article**

**Fusidic Acid Reverses Chemoresistance in Breast Cancer via Targeting DDX6 to Downregulate GSK-3β/β-catenin Signaling**

*Xiaxia Fan*, *Dan Guo*, *Songtao Li, Jinmiao Tian*, *Chaotong Zhang*, *Zhuoyu Li ^*^*

Xiaxia Fan, Dan Guo, Songtao Li, Jinmiao Tian, Chaotong Zhang, Zhuoyu Li

Key Laboratory of Chemical Biology and Molecular Engineering of the National Ministry of Education, Institute of Biotechnology, Shanxi University, Taiyuan 030031, China

E-mail: lzy@sxu.edu.cn; Tel: +86 351 7018268

**Supplemental Figure and Figure Legends**

**
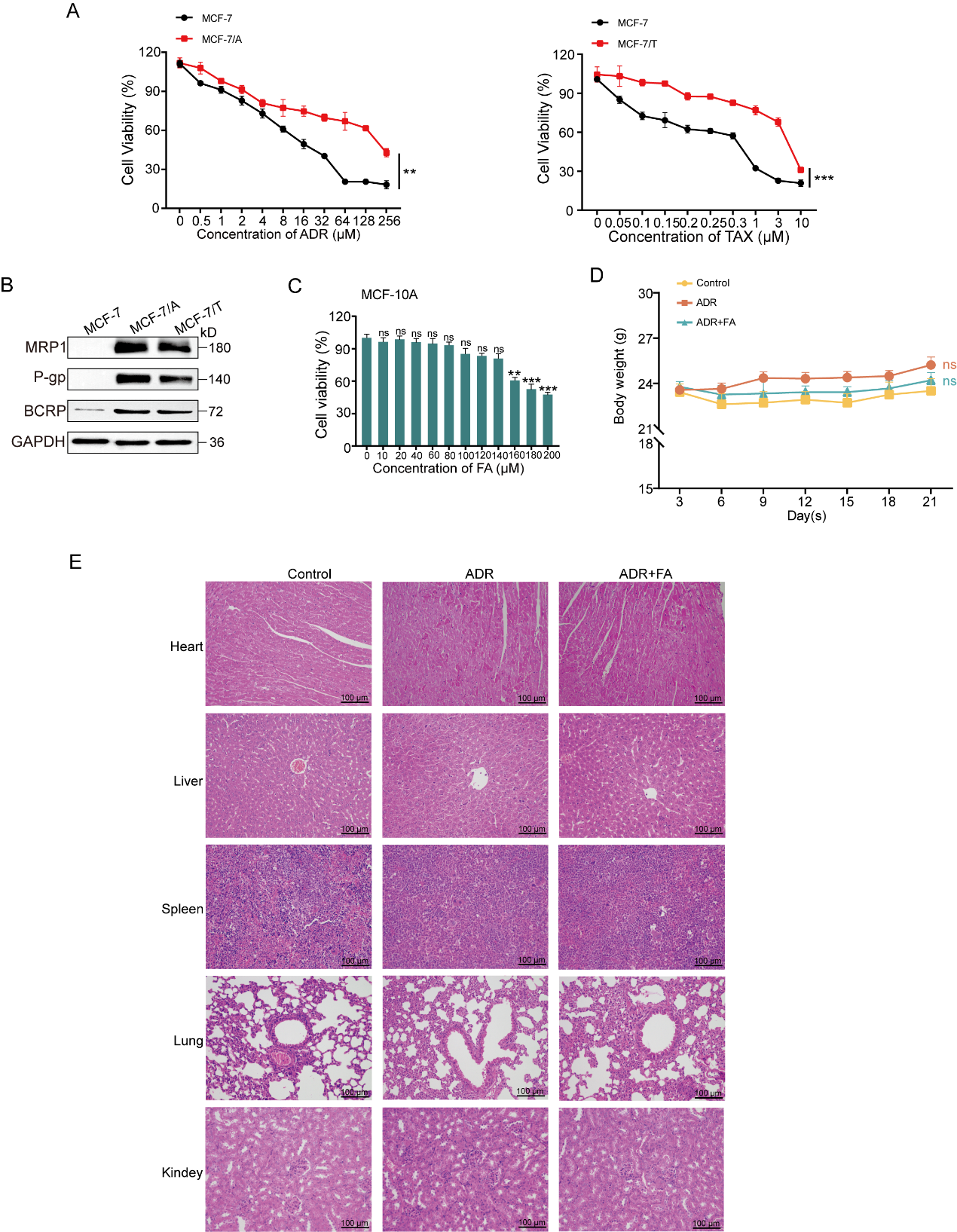
**

**Figure S1.** Safety monitoring analysis of the use of FA. (A) The cell viability of MCF-7/A, MCF-7/T, and MCF-7 cells was determined by Cell Counting Kit-8 (CCK8) after treatment with different concentrations of ADR or TAX for 48 h, n=3. (B) Protein levels of MRP1, P-gp, and BCRP were analyzed by immunoblotting in MCF-7/A, MCF-7/T, and MCF-7 cells, n=3. (C) Cell viability of MCF-10A cells was determined by CCK8 after treating cells with different concentrations of FA for 48 h, n=3. (D) Showing the effect of different treatments on the body weight of mice, n=6. (E) H&E staining analysis of the heart, liver, spleen, lungs, and kidneys of mice (scale bar, 100 μm), n=3. Data are presented as mean ± SEM (A, C, and D) and were analyzed using Student's t-test (A and D) or one-way ANOVA (C) with Bonferroni’s multiple comparisons test. ***p* < 0.01; ****p* < 0.001 versus MCF-7 cell. ns, not significant.


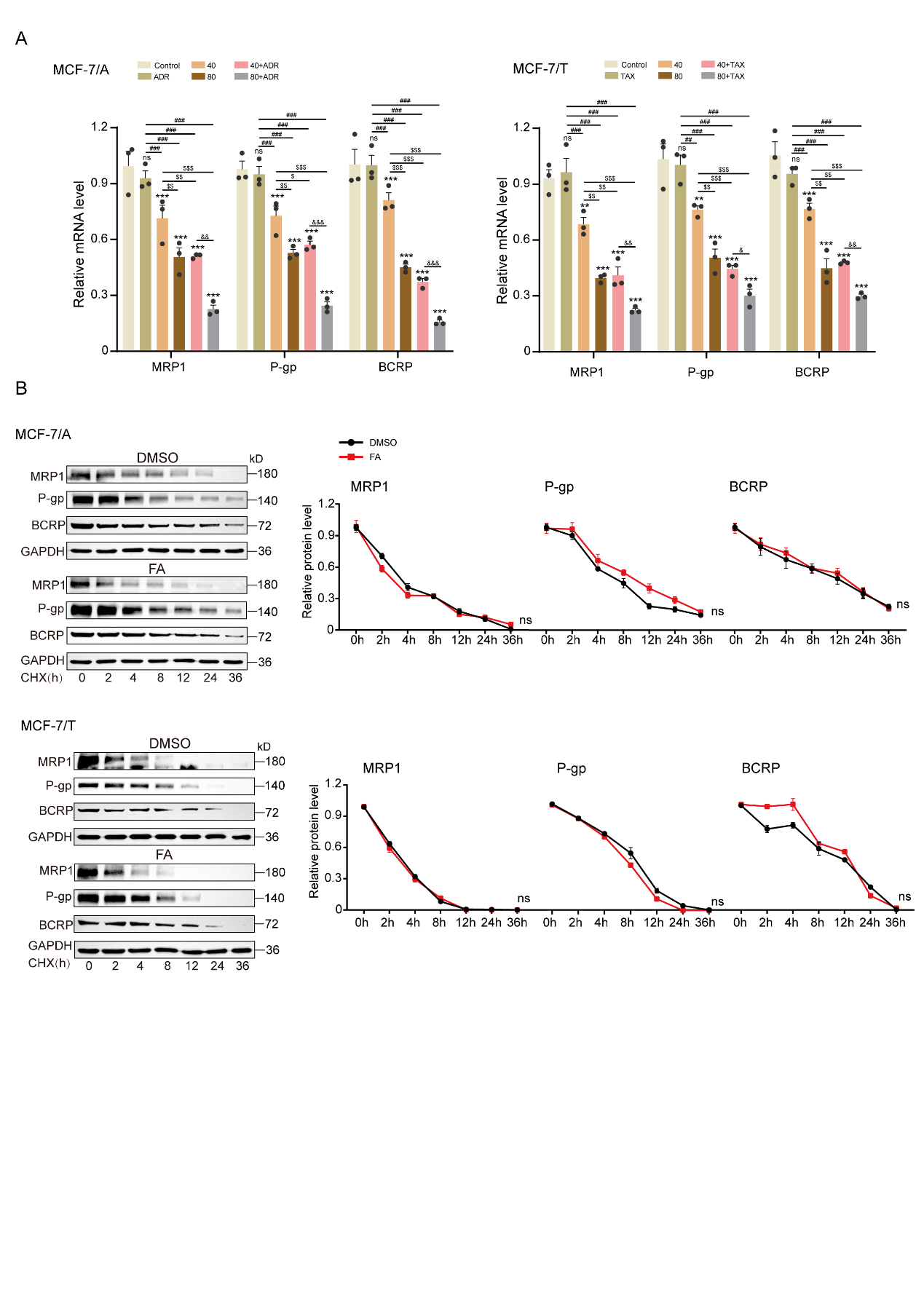


**Figure S2**. FA inhibits the expression of ABC transporter proteins to increase chemosensitivity. (A) Resistant cells treated with chemotherapeutic agents (ADR,32 μm /TAX,1μm) and FA (40, 80 μm) alone or in combination for 48 h, qPCR to analyze the expression of MRP1, P-gp, and BCRP, n=3. (B) MCF-7/A and MCF-7/T cells were treated with DMSO (control) and FA, and the half-life of MRP1, P-gp, and BCRP was determined by the CHX Chase assay. The quantitative results are shown below, n=3.

Data are presented as mean ± SEM (A and B) and were analyzed using Student's t-test (B) or one-way ANOVA (A) with Bonferroni’s multiple comparisons test. ***p* < 0.01; ****p* < 0.001 versus control. ##*p* < 0.01; ###*p* < 0.001 versus ADR or TAX-treated group; $*p* < 0.05; $$*p* < 0.01; $$$*p* < 0.001 versus 40 μm FA-treated group. &*p* < 0.05; &&*p* < 0.01; &&&*p* < 0.001 versus 40 μm FA in combination with ADR/TAX. ns, not significant.

**
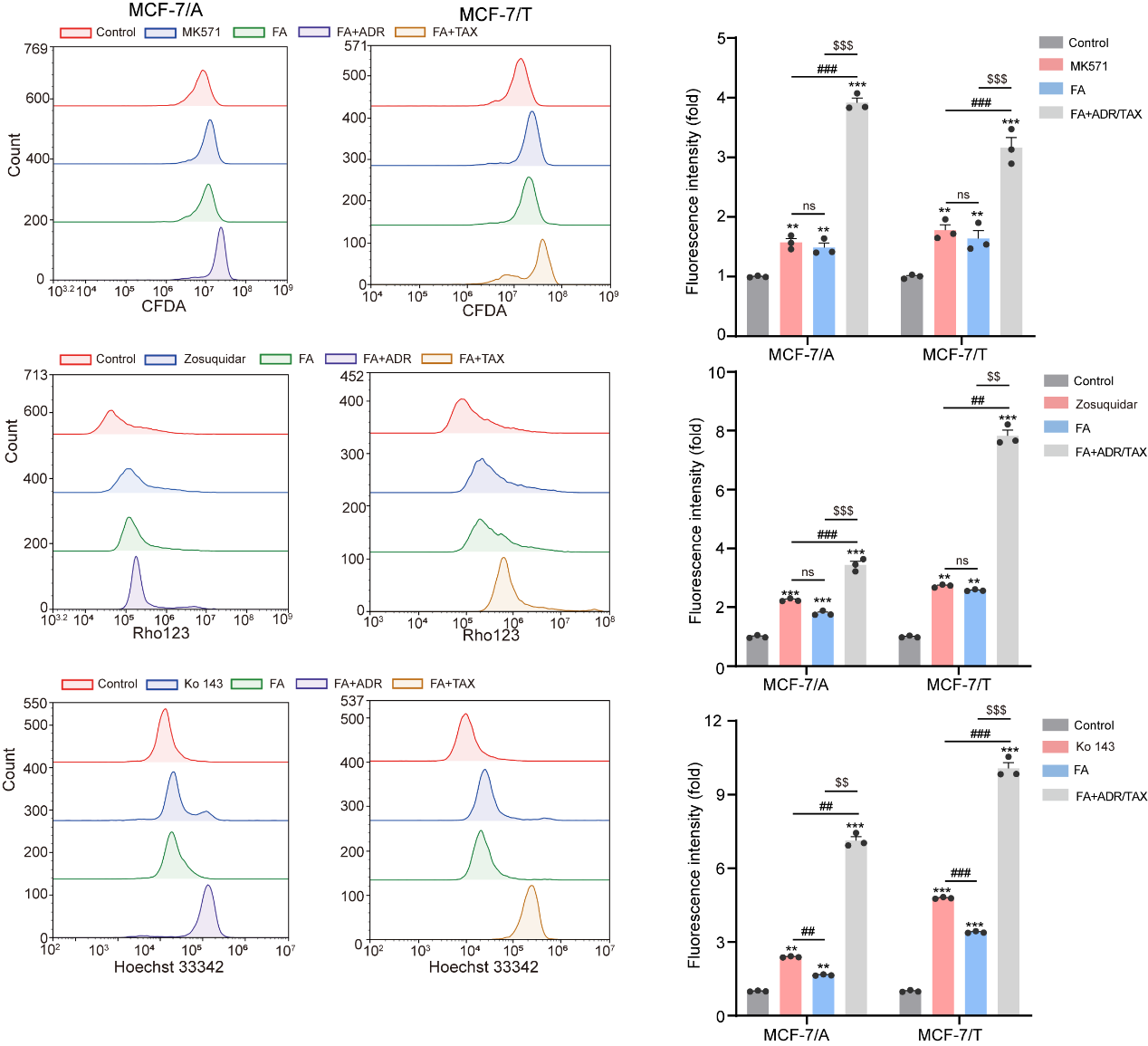
**

**Figure S3.** Comparative analysis of drug efflux by FA and ABC transporter protein inhibitors. Resistant cells were treated with inhibitors 20 μm MK571, 1 μm zosuquidar, and 10 μm Ko 143, and FA or FA in combination with ADR/TAX for 48 h. Fluorescence intensities of CFDA, Rho123, and Hoechst 33342 were analyzed by flow cytometry, n=3. Data are presented as mean ± SEM (A) and were analyzed using one-way ANOVA (A) followed by Bonferroni’s multiple comparisons test. ***p* < 0.01; ****p* < 0.001 versus control. ##*p* < 0.01; ###*p* < 0.001 versus MK571, zosuquidar and Ko 143 treated group; $$*p* < 0.01; $$$*p* < 0.001 versus 80 μm FA-treated group. ns, not significant.


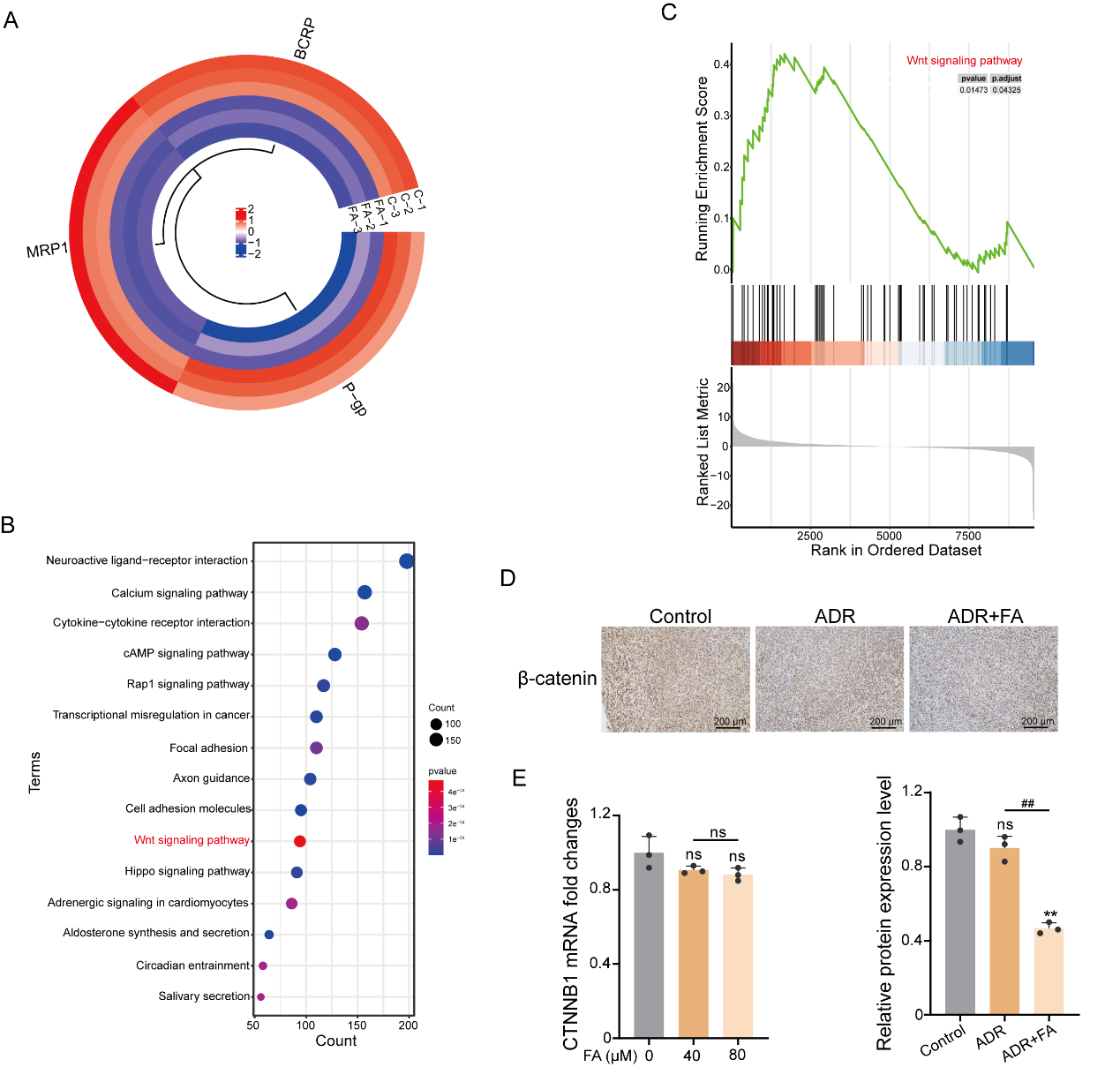


**Figure S4**. Analysis of the importance of the Wnt signaling pathway on chemotherapy resistance and the inhibitory effect of FA on β-catenin. (A) Heatmap of MRP1, P-gp, and BCRP expression in RNA sequencing. (B) KEGG analysis of differentially expressed genes in dataset GSE141698. (C) GSEA showed that the set of genes associated with the Wnt signaling pathway was significantly enriched among the differentially expressed genes in MCF-7 and MCF-7/A cells. NES, normalized enrichment score. FDR, false discovery rate. (D) Representative images of immunohistochemically stained β-catenin in tumor tissues (scale bar, 200 μm), n=3. (E) Analysis of the effect of FA on β-catenin mRNA levels by qPCR, n=3. Data are presented as mean ± SEM (D and E) and were analyzed using one-way ANOVA (D and E) followed by Bonferroni’s multiple comparisons test. ***p* < 0.01 versus control. ##*p* < 0.01 versus ADR-treated group. ns, not significant.


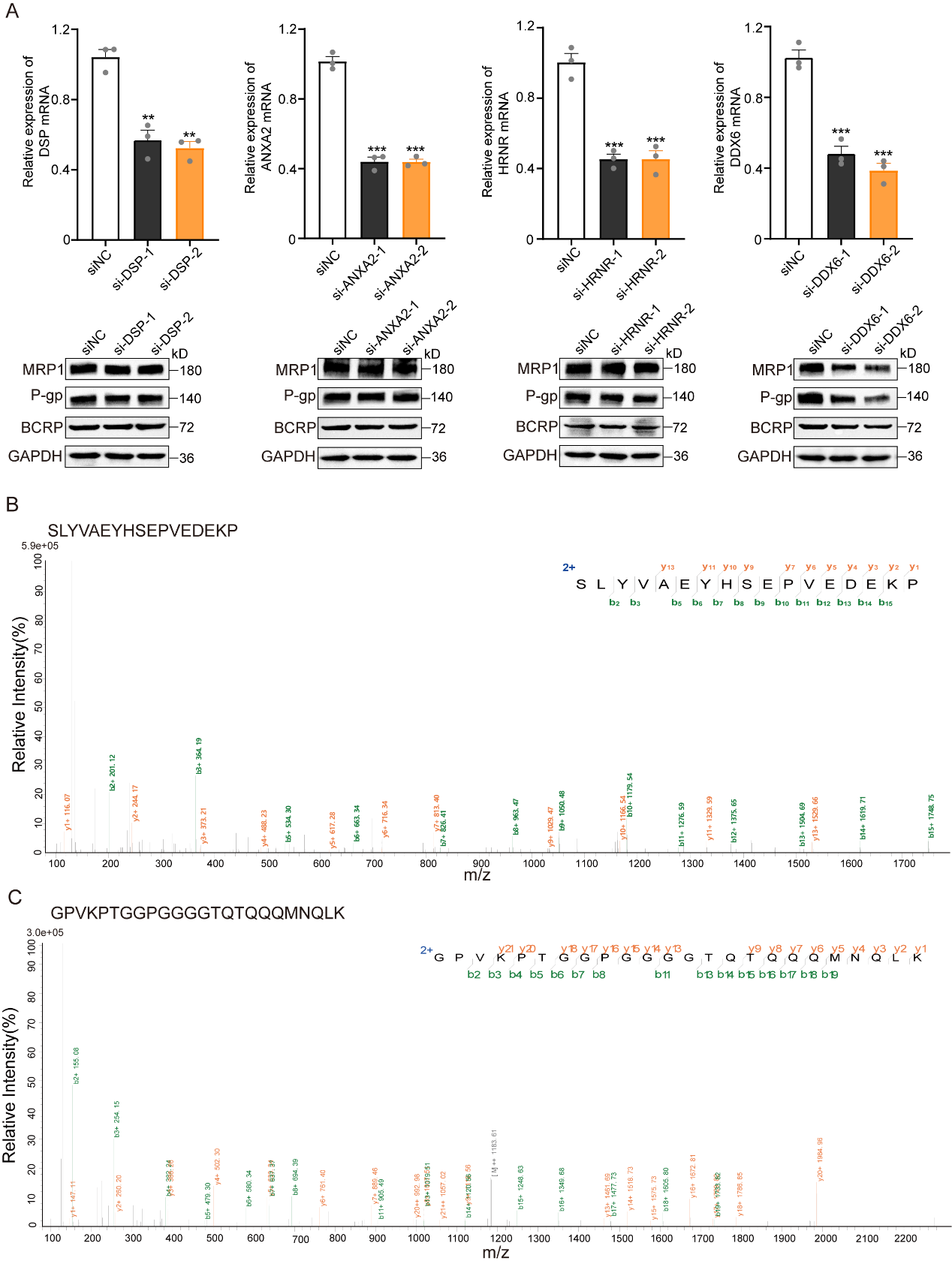


**Figure S5**. Identification and mass spectrometry analysis of the target protein DDX6 for FA. (A) MCF-7/A cells were transfected with siRNA of DSP, ANXA2, HRNR, and DDX6. qPCR was used to detect the knockdown efficiency, and immunoblotting was used to analyze the changes in the protein expression of MRP1, P-gp, and BCRP after the knockdown of DSP, ANXA2, HRNR, and DDX6, n=3. (B) Schematic of the secondary mass spectrum of DDX6 using Biotin-FA for Pulldown extraction. (C) Schematic of the secondary mass spectrum of DDX6 obtained using DARST. Data are presented as mean ± SEM (A) and were analyzed using one-way ANOVA (A) followed by Bonferroni’s multiple comparisons test. ***p* < 0.01; ****p* < 0.001 versus siNC group.


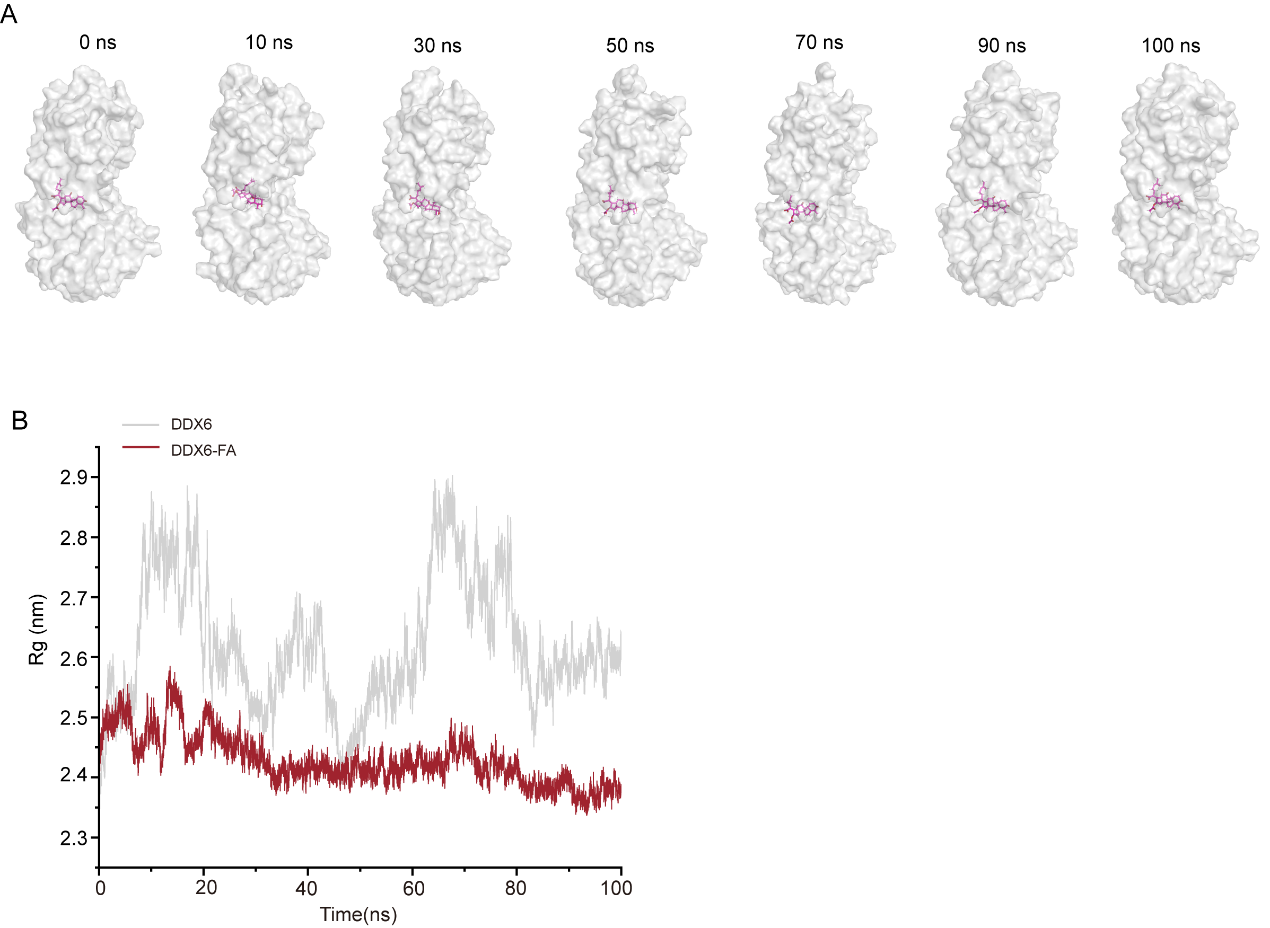


**Figure S6**: Validate the stability of the interaction between FA and DDX6. (A) The binding patterns between the small molecule and the protein (FA-DDX6) at different time points during the MD simulation. (B) Changes in the Rg of the DDX6 and FA-DDX6 complex during MD simulation.


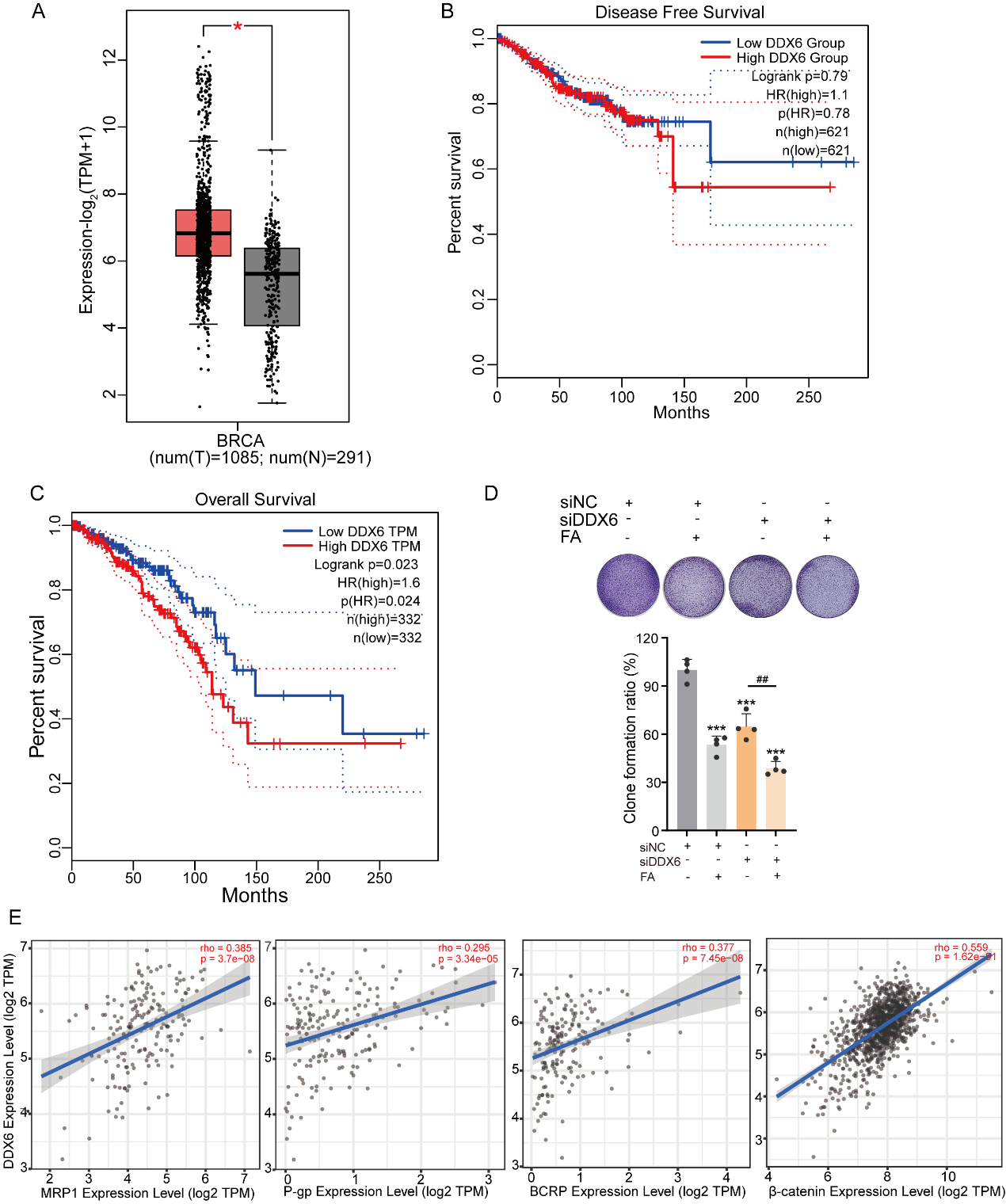


**Figure S7**. DDX6 is an oncogene and prognostic biomarker in BC. (A) Expression of DDX6 in BRCA (n = 1085) and normal breast tissue (n = 291) was analyzed by GEPIA. (B-C) Correlation between DDX6 expression and overall survival and disease-free survival of BRCA patients. (D) Cells knocked down for DDX6 or FA intervention were analyzed by colony formation assay (upper panel). Relative colony formation rates are expressed as SEM ± mean (lower panel), n = 4. (E) Correlation analysis of DDX6 with MRP1, P-gp, BCRP, and β-catenin. Data are presented as mean ± SEM (D) and were analyzed using one-way ANOVA (D) followed by Bonferroni’s multiple comparisons test. **p* < 0.05; ****p* < 0.001 versus cancerous tissues or siNC. ##*p* < 0.01 versus siDDX6.


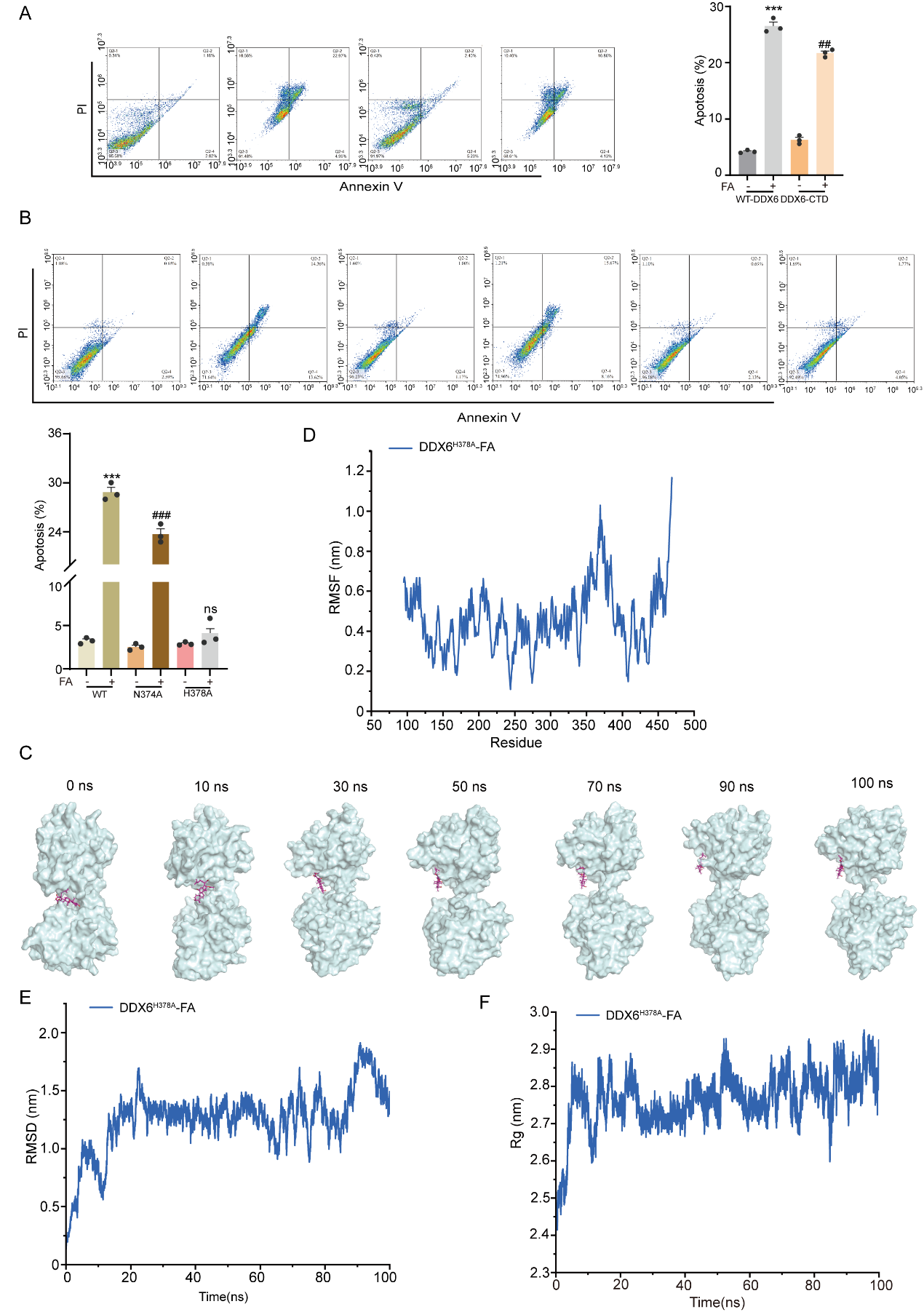


**Figure S8**. Exploring the amino acid sites where FA binds to DDX6. (A) Apoptosis of wild-type or CTD-truncated mutants of DDX6 treated with 80 μm FA for 48 h was measured by flow cytometry. Quantitative results are shown on the right, n=3. (B) Apoptosis was measured by flow cytometry in wild-type or N374A and H378A mutants treated with 80 μm FA, and quantitative results are shown below, n=3. (C) The binding patterns between the small molecule and the protein (FA-DDX6^H378A^) at different time points during the MD simulation. (D) RMSF was calculated based on MD simulation trajectories. (E) Time-dependent changes in the RMSD of the FA-DDX6^H378A^ complex during MD simulation. (F) Changes in the Rg of the FA-DDX6^H378A^ complex during MD simulation. Data are presented as mean ± SEM (A and B) and were analyzed using one-way ANOVA (A and B) followed by Bonferroni’s multiple comparisons test. ****p* < 0.001 versus transfected wild-type DDX6. ##*p* < 0.01; ###*p* < 0.001 versus CTD truncation mutants transfected with DDX6 or point mutations transfected with DDX6. ns, not significant.

**Supplemental table**

**Table S1. IC_50_ values of ADR and TAX in drug-resistant BC cells and MCF-7 cells**

| Chemotherapy | IC_50_ (μm ) | | Resistance index |
| --- | --- | --- | --- |
| drug | MCF-7 | MCF-7/MDR |  |
| ADR | 10.33±2.82 | 129.4±4.6 | 12.5 |
| TAX | 0.5±0.84 | 5.3±3.1 | 10.6 |

**Table S2. Primers used in qPCR analysis**

| **Gene** |  | **Sequence (5’-3’)** |
| --- | --- | --- |
| CTNNB1 | Forward | GGCTCTTGTGCGTACTGTCCTTC |
|  | Reverse | GCTTCTTGGTGTCGGCTGGTC |
| MRP1 | Forward | GCTGAGTTCCTGCGTACCTATGC |
|  | Reverse | GCCATTCTCCATTTGCTTTGCTTCC |
| P-gp | Forward | GATTGCTCACCGCCTGTCCAC |
|  | Reverse | CGTGCCATGCTCCTTGACTCTG |
| BCRP | Forward | GCAGCAGGTCAGAGTGTGGTTTC |
|  | Reverse | ACTGAAGCCATGACAGCCAAGATG |
| DSP | Forward | TGGCCTCATACACCTCAGGA |
|  | Reverse | CGAGCATGAACATCTGCAGC |
| ANXA2 | Forward | ACACCCCCAAGTGCATATGG |
|  | Reverse | TGCTGCGGTTGGTCAAAATG |
| HRNR | Forward | TGGATCTGGCCATTCCCCTA |
|  | Reverse | GAGACTCATATGGGCCACGG |
| DDX6 | Forward | AGAGCGGTGCCTACCTCATTCC |
|  | Reverse | CCTCCCATGTGTTTGCTGACCTG |
| GAPDH | Forward | ACAACTTTGGTATCGTGGAAGG |
|  | Reverse | GCCATCACGCCACAGTTTC |

**Table S3. Sequences of siRNAs against specific targets in this study**

| **Gene** | **Sequence (5’-3’)** |
| --- | --- |
| siDSP-1 | CGCCAGGAUUCCUUAGAAUTT |
| siDSP-2 | GGGAGAUCAUGUGGAUCAATT |
| siANXA2-1 | UUGCUGAUCGGCUGUAUGATT |
| siANXA2-2 | ACCAACCGCAGCAAUGCACTT |
| siHRNR-1 | GGCAGAGCUGAAAGAACUUTT |
| siHRNR-2 | GAACGACACGGAUCUAGCUTT |
| siDDX6-1 | GAGGCAGGAACAUCGAAAUTT+ |
| SiDDX6-2 | CACCAACACAAUCAAUAAUTT |

**Table S4. Antibodies used for immunoblotting**

| Name Source | |  | Dilution ratio | Catalog number |
| --- | --- | --- | --- | --- |
| rabbit anti-MRP1 | Proteintech | | 1:1000 | 27825-1-AP |
| rabbit anti-P-gp | Proteintech | | 1:1000 | 22336-1-AP |
| rabbit anti-BCRP | Proteintech | | 1:1000 | 27286-1-AP |
| mouse anti-β-catenin | Proteintech | | 1:1000 | 66379-1-Ig |
| rabbit anti-phospho-β-catenin | CST | | 1:1000 | 9561 |
| (Ser33/37/Thr41)  rabbit anti-phospho-β-catenin (Thr41/Ser45) | CST | | 1:1000 | 9565 |
| rabbit anti-phospho-GSK-3β(Ser9) | CST | | 1:1000 | 9336 |
| rabbit anti-DDX6 | Proteintech | | 1:1000 | 14632-1-AP |
| rabbit anti-HA | Proteintech | | 1:1000 | 51064-2-AP |
| rabbit anti-ki67 | Proteintech | | 1:1000 | 27309-1-AP |
| mouse anti-HSC70 | Bioss | | 1:100 | bsm-33211M |
| rabbit anti-Ubiquitin | Proteintech | | 1:1000 | 10201-2-AP |
| Mouse anti-GAPDH | Proteintech | | 1:20000 | 60004-1-Ig |
| Mouse anti-β-actin | Proteintech | | 1:20000 | 66009-1-Ig |
| HRP conjugated goat anti-rabbit IgG | Bioss | | 1:2000 | bs-0293R |
| HRP conjugated goat anti-mouse IgG | Bioss | | 1:2000 | bs-0293M |

**Table S5. Primers for the detection of constructed plasmids**

| Name | Sequence (5’-3’) |
| --- | --- |
| pCMV-DDX6 | F:ATCATGTCTGGATCCCCGCGGCCGCGGTACCTCGAGTCAAGGCTTCTCGTC |
|  | R:CGCTCTTATGGCCATGGAGGCCCGAATTCGGTCGACGATGTCCACCGCTAGGACAGAGAACCCT |
| pCMV-DDX6-127 | F:ATCATGTCTGGATCCCCGCGGCCGCGGTACCTCGAGTCAGATGGACTCCTCCTGGATGG |
|  | R:CGCTCTTATGGCCATGGAGGCCCGAATTCGGTCGACGATGAGCACAGCCAGGACAGAGAA |
| pCMV-DDX6-NTD | F:ATCATGTCTGGATCCCCGCGGCCGCGGTACCTCGAGTCACTTCAGTGTCAGCTCCT |
|  | R:CGCTCTTATGGCCATGGAGGCCCGAATTCGGTCGACGATGTCCACAGCCAGAACAGAGAACCCCGT |
| pCMV-DDX6-CTD | F:ATCATGTCTGGATCCCCGCGGCCGCGGTACCTCGAGTCAGGGCTTCTCGTCCTCCACA |
|  | R:CGCTCTTATGGCCATGGAGGCCCGAATTCGGTCGACGGGAGTGACCCAGTACTACGCCTACGTGA |
| pCMV-DDX6  (G143A) | F:TTATGGCCATGGAGGCCCGAATTCGGTCGACgATGTCCACCGCTAGGACAG |
|  | R:GTCTGGATCCCCGCGGCCGCGGTACCTCGAGTCAAGGCTTCTCGTCCTC |
| pCMV-DDX6  (N374A) | F:TTATGGCCATGGAGGCCCGAATTCGGTCGACgATGTCCACCGCTAGGACAG |
|  | R:GTCTGGATCCCCGCGGCCGCGGTACCTCGAGTCAAGGCTTCTCGTCCTC |
| pCMV-DDX6 | F: TTCCAGCAATGTAATTCAGT |
| (H378A) | R: TAAGCCATTGGATCTATCAC |
| pX330-DDX6-sgRNA | ATGGACTATCATATGCTTACCGTA |
